# A comprehensive review of crop stress detection: destructive, non-destructive, and ML-based approaches

**DOI:** 10.3389/fpls.2025.1638675

**Published:** 2025-09-05

**Authors:** Aman Muhammad, Zahid Ullah Khan, Javed Khan, Abdul Sattar Mashori, Aamir Ali, Nida Jabeen, Ziqi Han, Fuzhong Li

**Affiliations:** ^1^ College of Agricultural Engineering, Shanxi Agricultural University, Taigu, Jinzhong, China; ^2^ School of Software, Shanxi Agricultural University, Taigu, Jinzhong, China; ^3^ College of Information and Communication Engineering, Harbin Engineering University, Harbin, China; ^4^ Department of Software Engineering, University of Science and Technology Bannu, Bannu, Pakistan; ^5^ College of Agriculture, Shanxi Agricultural University, Taigu, Jinzhong, China; ^6^ School of Communications and Information Engineering, Chongqing University of Posts and Telecommunications, Chongqing, China

**Keywords:** crop stress types, stress analysis, destructive analysis techniques, non-destructive analysis techniques, machine learning analysis

## Abstract

Agriculture stands as a foundational element of life, closely linked to the progress and development of society. Both humans and animals depend on agriculture for a wide range of essential services, such as producing oxygen and food, along with vital raw materials for clothing, medicine, and other necessities. Given agriculture’s vital role in supporting individual well-being and driving global progress, protecting and ensuring the long-term sustainability of agriculture is essential. This is crucial for securing resources and maintaining environmental balance for future generations. In this context, in our review we have examined the various factors that can interfere with the normal physiological and developmental functions of plants and crops. These factors, referred to scientifically as stressors or stress conditions, include a wide range of both biotic and abiotic challenges. In this work we have systematically addressed all the major categories of stress that plants may encounter throughout their lifecycle. Additionally, because plants tend to exhibit recognizable physiological or biochemical responses to stress, we have cataloged the associated stress indicators. These indicators were identified through various assessment techniques, including both destructive and non-destructive approaches. A significant advancement highlighted in our review is the integration of Machine Learning (ML) algorithms with non-destructive methodologies, which has substantially enhanced the accuracy, scalability, and real-time capability of plant stress detection. These ML-enhanced systems leverage high-dimensional data acquired through remote sensing modalities, such as hyperspectral imaging, thermal imaging, and chlorophyll fluorescence. These ultimately help in enabling the early identification of biotic and abiotic stress signatures. Through advanced pattern recognition, feature extraction, and predictive modeling, ML facilitates proactive anomaly detection and stress forecasting, thereby mitigating yield losses and supporting data-driven precision agriculture. This convergence represents a significant step toward intelligent, automated crop monitoring systems. Finally, we conclude the article with a concise discussion of the potential positive roles that certain stress conditions may play in enhancing plant resilience and productivity.

## Introduction

With time, the human population has continued to grow, along with the number of farm animals such as chickens, cows, camels, and others. According to the data from the United Nations (UN), the current human population is approximately 8.2 billion, while the exact number of farm animals remains uncertain and can only be estimated (Worldometer, 2024). To meet fundamental needs such as nutrition, shelter, clothing, medicine, energy, oxygen production, water cycle regulation, livestock feed, and raw materials for industry, plants and crops play a central role. They serve as the primary source of essential resources that are crucial for sustaining life and supporting the survival of all living beings (Marchev et al., 2021; Brown et al., 2022; Jørgensen et al., 2022). However, plants and crops are persistently exposed to a wide array of external and internal stressors. These stress-inducing factors impact their physiological activities, growth, development, and overall survival. Stress is widely described as any adverse occurrence or scenario that has a negative impact on a plant’s metabolism.

Stress factors also known as stressors, arise from a combination of human-induced activities such as industrialization, deforestation, pollution, and unsustainable farming practices, along with those originating from natural processes such as droughts, floods, pests, and diseases. These collectively impose significant pressure on agricultural systems. For instance, the impacts of global warming (Lesk et al., 2021; Zandalinas et al., 2021; Singh et al., 2022) have led to noticeable alterations in rainfall patterns across different regions of the world, causing either an increase or decrease in precipitation levels. These shifts influence other climatic variables that are associated with plant and crop stress. This includes fluctuations in temperature patterns, variations in humidity levels, and changes in wind strength and direction (Grossman, 2023). Warmer and humid conditions create a favorable environment for pathogens to reproduce with quick successions, which can cause stress in plants and crops. In this condition, plants must adapt to these shifting environmental conditions through physiological processes, ultimately leading to decreased overall yield. Furthermore, the intricate interplay between multiple abiotic and biotic stressors often results in tradeoffs; enhancing tolerance to one stressor may inadvertently increase a plant’s vulnerability to another stress (Kopecká et al., 2023). Due to the exposure to such circumstances that are unfamiliar and related to stress factors, these will be considered a hazard for the adopted genotypes. Additionally, the unfortunate reduction in available fertile land, along with the evolution of pests and pathogens that have become more resistant to pesticides and herbicides, further exacerbates the challenges faced by modern agriculture. These specific reasons have made the study of how crops and plants respond to these evolving stress factors very important in the recent past (Kashyap and Kumar, 2021). To counteract the aforementioned reasons, a careful discernment of the responses by plants to stress is now inevitable. This knowledge is essential for developing such crop varieties that will be capable of withstanding rapidly changing climatic conditions. However, with time, plants and crops have also evolved morphologically, physiologically, biochemically, and in terms of molecular operational adaptation levels. This has made them capable of handling these stressors by sensing them at an appropriate time and taking the correct measures against them (Chakraborti et al., 2022; Zahedi et al., 2025). Plants and crops have evolved very intricate communication networks within their bodies that are based on hormone signaling, the use of metabolites and stress protein responsiveness. All these evolutionary skill sets that are developed by crops and plants enable them to withstand or adapt to upcoming unknown stressful conditions either from humans or nature itself (Ashraf et al., 2018; Mansour and Hassan, 2022).

In the past, assessing plant stress primarily involved destructive methods, which required harvesting plant tissues to analyze their physiological and biochemical attributes. Techniques such as chlorophyll extraction, measuring leaf water potential, and evaluation of enzyme activities or hormone levels in the laboratory were commonly employed (Galieni et al., 2021). While these methods provided precise results, they were often labor-intensive, time-consuming, and unsuitable for large-scale agricultural monitoring. With advancements in sensing technologies, non-destructive methods have become increasingly favored. Approaches such as hyperspectral imaging, thermal infrared sensing, and fluorescence detection now enable researchers to monitor stress in real time without harming the plants (Ang and Lew, 2022).

Moreover, remote sensing technologies, including drones, Unmanned Aerial Vehicles (UAVs), satellites, and ground-based sensor networks allow for the collection of vast amounts of data across extensive agricultural regions, offering continuous, high-resolution insights into plant health (Dong et al., 2024). The application of Machine Learning (ML) has further enhanced the capabilities of non-destructive techniques by enabling the automated detection, classification, and prediction of plant stress (Gill et al., 2022). By training ML models on datasets consisting of spectral signatures, thermal patterns, or image-based features, these systems can accurately identify different stress conditions such as drought stress, nutrient deficiencies, and disease infections, and even forecast stress development before visible symptoms manifest (Elvanidi and Katsoulas, 2022a; Rico-Chávez et al., 2022). Unlike previous reviews, this work emphasizes the synergy between ML algorithms and remote sensing techniques in enabling real-time, scalable plant stress assessment.

**Figure 1:**
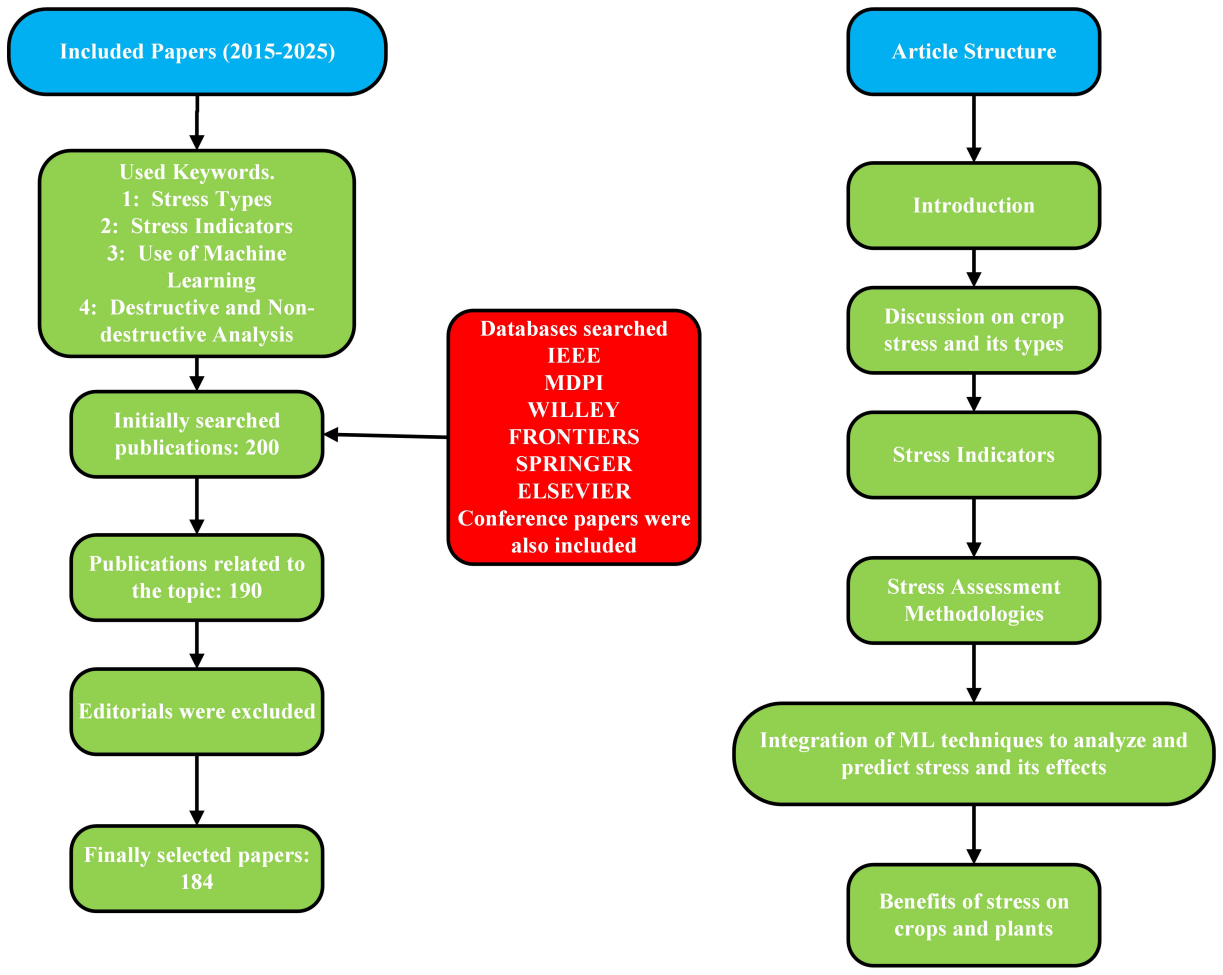
Flowchart Of Our Article And Adopted Research Methodology.

The remainder of this article is organized as follows: section 2 presents a comprehensive analysis of the biotic and abiotic stresses. A discussion on the stress indicators that are caused by the stress factors is provided in section 3. In section 4 we have included a brief discussion of destructive and non-destructive stress analysis techniques. The readers will find a discussion on the use of ML algorithms in section 5. These algorithms are integrated with non-destructive stress analysis methods to enhance precision and forecast future occurrences. In section 6 we have discussed the benefits of stress to the plants and crops. **Figure 1** offers a clear and comprehensive flow diagram outlining the search strategy employed for this article.

## Types of crop stress

**Figure 2:**
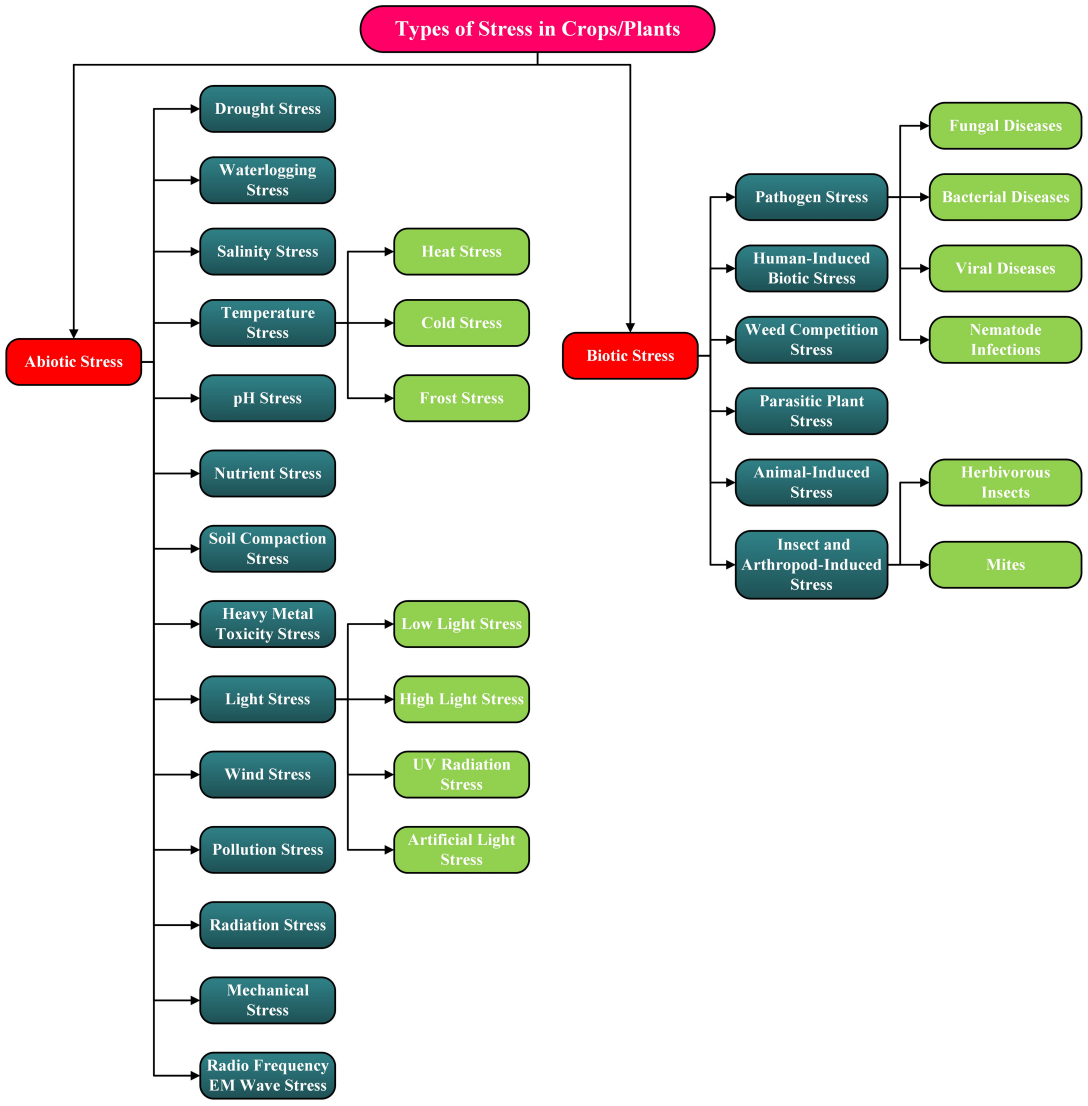
Flow diagram illustrating the various types of stress, or stressors, experienced by crops and plants. These stressors are categorized as abiotic or biotic.

Crop/Plant stress is defined as any external condition that may adversely affect a plant’s growth and productivity. These stressors are generally divided into two main types: abiotic, resulting from environmental or physical causes, and biotic, resulting from living organisms. This section offers an in-depth exploration of the main categories and subcategories of plant stress. To enhance the readers’ comprehension, **Figure 2** features a detailed flow diagram illustrating the classification of these stress factors.

### Abiotic stress

Abiotic stress on crops and plants is defined as the effects of the conditions that are centric to the environment excluding those caused by living organisms. Very common types of this stress type include the effects due to extreme changes in temperature. These can trigger drought, which in turn increases the salinity of the soil in some specific cases. Many more types of abiotic stress will be discussed in the next subsection, where readers will find brief discussions on each type.

#### Drought stress

Drought is a natural phenomenon that occurs when water is not available. This water can be in the form of rain, running rivers, dammed waters, or groundwater. Whenever drought hits a region, we can see that it detrimentally affects plant growth, which in turn has an adverse effect on yield. Drought-affected areas are essentially disturbed in homeostasis, which interferes with crucial physiological and biochemical functions. Key impacts of stress on crops and plants due to drought include diminished cell turgor, stomatal closure, and suppressed photosynthetic activities due to restricted CO_2_ uptake. Drought stops the uptake of the nutrients and produces a hormonal imbalance, which is the reason for increased levels of Abscisic Acid (ABA) (Reinelt et al., 2023; Tian et al., 2025). Drought also has the tendency to increase Reactive Oxygen Species (ROS), which causes oxidative damage. Various researchers have found that all of the aforementioned phenomena caused by drought (whether recent or ongoing) will disrupt biomass production, hinder reproductive processes, and cause substantial yield declines. However, it has been found that, over time, plants have also evolved to counteract the effects of drought, including through morphological alterations such as increased root-to-shoot ratio, osmotic regulation through the buildup of compatible solutes, such as proline and glycine betaine (Ozturk et al., 2021; Chachar et al., 2025) and the activation of drought-responsive genetic pathways that enhance tolerance.

#### Flooding/waterlogging stress

Waterlogging and flooding are critical environmental stressors that interfere with vital physiological and metabolic activities in plants. When soils become saturated with water, air is expelled from soil pores, creating oxygen-deficient conditions (hypoxia or anoxia) that severely impair aerobic respiration in the root zone (Zahra et al., 2021). This lack of oxygen compromises mitochondrial Adenosine Triphosphate Production (ATP), thereby disrupting energy-dependent processes such as nutrient and ion transport. Prolonged anaerobic conditions may result in stunted root development (hypoplasia), the formation of air-filled cavities in roots (aerenchyma), and, in severe scenarios, root tissue death (necrosis) (Teoh et al., 2022). Furthermore, researchers have mentioned that saturation also alters the soil’s redox balance, which influences the solubility of essential nutrients. This, in turn, typically causes deficits in the nitrogen, phosphorus, and potassium absorption, while at the very same time increasing the consolidation of potentially toxic elements such as manganese and iron. Toxic effects due to ethanol, methane and lactic acid can also be seen in such scenarios as these are byproducts of the flooded soils. The buildup of ethylene causes various aging-related symptoms that are readily seen in the leaves of crops and plants. Initially, yellowing of the leaves is one symptom; however, shedding and shoot elongation can also be observed (Gu et al., 2021). However, with the advancements in findings on this subject over time, it has also been concluded that species tolerance, growth stage, flood duration, and soil characteristics also play a critical role.

#### Salinity stress

Salinity stress is a critical abiotic factor that significantly affects a plant’s growth, development, and productivity by interfering with key physiological, biochemical, and molecular functions (Sahito et al., 2024). Elevated levels of soluble salts, particularly sodium chloride (NaCl) decrease the soil’s water potential in the rhizosphere. This leads to osmotic stress, which impedes root water uptake even when soil moisture is present. This osmotic stress disrupts cellular turgor, limits cell elongation, and ultimately restricts overall plant development. Concurrently, ionic stress occurs when excess sodium (Na^+^) and chloride (Cl^−^) ions accumulate in plant tissues, disrupting enzymatic processes, destabilizing cell membranes, and impairing metabolic pathways (Shaikh et al., 2022; Yuan et al., 2024). Various researchers have mentioned that an increased concentration of sodium in the soil has the tendency to disrupt the digestion of important nutrients. These nutrients include potassium (K^+^), calcium (Ca^2+^), and magnesium (Mg^2+^). This imbalance of nutrients in crops and plants can result in deficiencies. However if we take a closer look at the cellular level, we will find out that an increase in salinity also tends to activate oxidative stress ROS, which results in lipid membrane degradation, protein malfunction, and genetic material damage. Some obvious signs of an increase in salinity include chlorosis, necrosis, premature leaf drop, and reduced overall biomass. However, the literature tells us that the severity of the aforementioned stress is also dependent on the tolerance of a species to the stressed environment, its growth stage, the type of salt accumulated in the soil, its concentration per square meter, and the duration of exposure. Overall, it is observed that salinity-induced stress on crops and plants has more significant effects in arid regions, sometimes becoming a major obstacle to the food security of people dependent on indigenous farming techniques (Ehtaiwesh, 2022).

#### Temperature stress

Temperature extremes encompassing heat, cold, and frost are significant stressors that detrimentally affect the physiological, biochemical, and molecular functions of plants, ultimately limiting their growth, development, and productivity. Heat stress occurs when ambient temperatures exceed the optimal range for a specific plant species, leading to impaired photosynthesis through destabilization of the photosystem. Heat stress compromises membrane fluidity, elevates respiration rates, and disrupts protein folding mechanisms, which collectively reduce carbon assimilation and disturb metabolic homeostasis (Saddhe et al., 2021). To counteract thermal damage, plants activate protective responses such as producing Heat Shock Proteins (HSPs), which aid in stabilizing and refolding damaged proteins. In contrast, cold and frost stress occur when temperatures drop below a plant’s tolerance limit, causing membrane rigidity, disruption of the cytoskeleton, and formation of extracellular ice. These lead to cellular dehydration and mechanical injury. Frost damage worsens when ice crystals form intracellularly, rupturing cells and causing permanent tissue damage. Cold stress has been observed to abolish and slow down the metabolism of the vegetation, affecting very critical stages of crops and plants, such as germination, flowering, and fruit development. Various researchers have shown that both increase and decrease in temperature activate oxidative stress. This is done through the accumulation of ROS, which in turn leads to damaging the crops and plants at the cellular level. Over time, however, some plant species have developed the capacity to lessen these effects by incorporating cryoprotectants, such as antifreeze proteins and osmolytes. However the exact effect of the severity of fluctuating temperature-based stress on vegetation whether high or low, is mainly determined by the growth stage, duration, and intensity of exposure. This can sometimes severely impair crop productivity and resilience (Ding and Yang, 2022).

#### Light stress

Moderate light stress, which includes low light levels (reduced photon flux), excessive light (high irradiance), and ultraviolet radiation, represents a stressor that disrupts a plant’s photosynthesis. In low-light scenarios, the limited supply of Photosynthetically Active Radiation (PAR) hampers chlorophyll’s ability to absorb photons, thereby diminishing electron transport, lowering ATP and Nicotinamide Adenine Dinucleotide Phosphate (NADPH) production, and constraining carbon fixation via the Calvin cycle. These effects lead to reduced plant growth and biomass (Roeber et al., 2021). Conversely, high light exposure causes excessive excitation of chlorophyll, leading to photoinhibition. This energy surplus promotes the generation of ROS, which cause oxidative damage to thylakoid membranes, proteins, and nucleic acids. It is observed that Ultraviolet B (UV-B) radiation exacerbates stress in plant cells. This occurs due to Deoxyribonucleic Acid (DNA) damage, protein cross-linking, lipid peroxidation, and disruption of hormone signaling pathways. However, with the passage of time plants have developed a protective mechanism to counteract this effect. This includes Non-Photochemical Quenching (NPQ), activation of the xanthophyll cycle, and increased activity of antioxidant enzymes such as Superoxide Dismutase (SOD), Catalase (CAT), and Ascorbate Peroxidase (APX), along with the accumulation of Ultra Violet (UV)-screening secondary metabolites such as flavonoids (Yang et al., 2019). However it has been observed that the effects of stress due to light exposure vary greatly depending on the presence of a specific photoreceptor, developmental stage and exposure duration.

#### Nutrient stress

Nutrient imbalances, arising from either deficiencies or toxicities, are significant stress factors that interfere with essential physiological activities, suppress plant growth, and lower agricultural productivity. The inadequate availability of vital macronutrients (e.g. nitrogen, phosphorus and potassium) or micronutrients (e.g. iron, zinc and boron) can disrupt primary metabolic pathways such as photosynthesis, respiration, and protein synthesis (Abbas et al., 2021). Deficiency symptoms commonly include chlorosis, necrosis, reduced meristem activity, and impaired reproductive development. Conversely, nutrient toxicity occurs when element concentrations exceed optimal thresholds, causing ionic imbalances, membrane disintegration, and oxidative damage due to the overproduction of ROS. Excesses of toxic elements such as manganese, aluminum, or sodium tend to block or disrupt the enzymatic functionality, assimilation of nutrients and osmotic regulation. Therefore, nutrient deficiency stress ultimately lessens cellular homeostasis, alters hormonal signaling, and heightens susceptibility to pests and environmental stress (Kumari et al., 2022). These issues are often intensified by suboptimal soil conditions, such as nutrient-poor soils, incorrect pH levels, salinity, or overfertilization. Effective nutrient management through soil analysis, customized fertilization plans, and comprehensive fertility practices is essential for maintaining nutrient balance and supporting sustainable crop production. In **Table 1**, we have summarized the effects caused by both the deficiencies and toxicities arising from the lack of various essential nutrients in crops and plants.

**Table 1:** Effects of nutrient deficiency and toxicity in plants.

S. no	Nutrient	Deficiency symptoms	Toxicity symptoms	References
1	Nitrogen (N)	Pale yellowing of older leaves, stunted growth, early leaf drop	Dark green foliage, leaf tip burn, delayed flowering	(Wani et al., 2022; Abbas et al., 2021)
2	Phosphorus (P)	Dark green or purplish leaves, stunted growth, poor root development	Leaf necrosis, interference with micronutrient uptake	(Abbas et al., 2021; Fathi and Afra, 2023)
3	Potassium (K)	Yellowing and browning of leaf edges, weak stems, poor fruit quality	Leaf burn, reduced magnesium and calcium uptake	(Li et al., 2022; Huang et al., 2022)
4	Calcium (Ca)	Blossom end rot in fruits, distorted young leaves, poor root growth	Leaf tip burn, interference with magnesium and potassium uptake	(De Bang et al., 2021; da Silva et al., 2021)
5	Magnesium (Mg)	Interveinal chlorosis in older leaves, leaf curling, premature leaf drop	Rare; can cause calcium deficiency	(Ishfaq et al., 2022; Chaudhry et al., 2021)
6	Sulfur (S)	Yellowing of young leaves, stunted growth, delayed maturity	Leaf necrosis, reduced uptake of other nutrients	(Zenda et al., 2021) (Dawar et al., 2023)
7	Iron (Fe)	Interveinal chlorosis in young leaves, stunted growth	Bronze coloration of leaves, reduced phosphorus uptake	(Merry et al., 2022; Zuluaga et al., 2023)
8	Manganese (Mn)	Interveinal chlorosis with brown spots, poor photosynthesis	Black spotting, brown necrotic areas, inhibited root growth	(Rai et al., 2021; Dey et al., 2023)
9	Zinc (Zn)	Stunted growth, shortened internodes, small leaves, chlorosis	Leaf chlorosis, inhibits root development	(Khan et al., 2022; Hamzah Saleem et al., 2022)
10	Copper (Cu)	Leaf curling, chlorosis, dieback of stems and twigs	Leaf chlorosis, reduced seed germination	(Moreira et al., 2022; Abbas et al., 2021)
11	Boron (B)	Death of growing points, brittle leaves, poor fruit/seed set	Leaf burn, thickened and brittle stems	(Bolaños et al., 2023; Botelho et al., 2022)
12	Molybdenum (Mo)	Yellowing of older leaves, leaf edge necrosis, poor nitrogen fixation	Rare; can induce copper deficiency	(Roychoudhury and Chakraborty, 2022; Banerjee and Nath, 2022)
13	Chlorine (Cl)	Wilting, chlorosis, necrosis of leaf tips	Leaf burn, reduced root growth, nutrient imbalances	(Abbas et al., 2021; Lilay et al., 2024)

#### Soil compaction stress

Soil compaction is a stress that impairs plant physiological processes and reduces crop yields by altering the physical integrity of the soil. It leads to increased bulk density and decreased porosity, which restricts root expansion, slows down seedling emergence, and obstructs the movement and absorption of water, air, and essential nutrients. Reduced oxygen availability results in hypoxic stress, which can disrupt the respiratory system in roots. This, in turn, has a direct weakening effect on microbial activity, which is essential for effective nutrient cycling (Shaheb et al., 2021). Additionally, compacted soils exhibit limited water infiltration and drainage, which promotes surface runoff, exacerbates erosion, and increases crop susceptibility to moisture extremes such as drought and waterlogging. In compacted soil conditions, it is common to observe the formation of a dense subsoil layer, due to which roots can lose their ability to penetrate and reach the moisture and nutrient reserves that are deep within the soil (Jamali et al., 2021). The primary causes of compaction include anthropogenic activities such as the use of heavy machinery, frequent tillage, and overgrazing. To address the negative effects of soil compaction, strategies such as implementing controlled traffic farming, adopting reduced tillage practices, incorporating cover crops, and utilizing deep-rooted plant species can improve soil structure and promote greater ecosystem resilience.

#### Heavy metal toxicity stress

Heavy metal toxicity is a critical stressor that negatively impacts the physiological and molecular functioning of plants and crops. Harmful metals such as cadmium (Cd), lead (Pb), arsenic (As), and mercury (Hg) frequently enter the soil through human-driven activities, including industrial waste release, mining operations, and the excessive application of agricultural chemicals. These metals are notable for their environmental persistence and pronounced ability to bioaccumulate in plant tissues (Ghuge et al., 2023). Once taken up by roots, heavy metals interfere with cellular homeostasis by disrupting nutrient transport mechanisms, altering enzyme activities, and compromising membrane stability. At the intracellular level, heavy metal stress provokes the excessive generation of ROS, resulting in oxidative damage that targets lipids, proteins, and nucleic acids. These disruptions negatively affect vital physiological processes such as photosynthesis, mitochondrial respiration, and hormonal signaling, especially pathways regulated by ABA and ethylene. Additionally, heavy metal exposure influences the expression of the genes related to metal sequestration, antioxidative responses, and detoxification systems, including the synthesis of phytochelatins and metallothioneins (Ali and Gill, 2022). Collectively, these effects lead to inhibited growth, developmental anomalies, and reduced agricultural productivity, while posing serious risks to food safety and ecological health.

#### pH stress

Soil pH imbalance is a stressor that negatively influences plant physiological functions and diminishes crop yields. When the pH strays from the optimal range of 5.5 to 7.5, typical for the majority of crops, nutrient solubility and uptake are impaired, leading to inefficient ion transport and disturbances in vital metabolic pathways. However the proliferation of cells and growth of crop and plant roots is severely hampered in acidic soils (pH < 5.5), due to the presence of aluminum (Al³^+^) and manganese (Mn^2+^). On the other hand, alkaline conditions (pH > 7.5) limit the availability of essential micronutrients such as iron (Fe^2+^), zinc (Zn^2+^), and copper (Cu^2+^), often resulting in visible symptoms of nutrient stress, such as interveinal chlorosis and reduced photosynthetic performance (Tsai and Schmidt, 2021). Additionally, soil acidity has been observed to affect microbial activities that are beneficial to the plants by disturbing their movement in the rhizosphere. This ultimately hinders the plant’s ability to better and more precise nitrogen fixation and organic matter decomposition. The effectiveness of agrochemicals also decreases under such conditions. Therefore researchers have used techniques to adjust the pH of soil using agricultural lime (CaCO_3_) or elemental sulfur (S), which have been found to be vital for restoring soil health, nutrient dynamics, and crop tolerance to pH-related stress (Gao et al., 2025).

#### Wind stress

Aerodynamic stress resulting from both excessive and limited wind exposure can significantly disrupt a plant’s architecture. Strong winds impose mechanical stress, leading to structural damage such as stem lodging, leaf fragmentation, and heightened water loss due to accelerated transpiration via stomatal and cuticular surfaces. These effects are exacerbated under drought conditions when elevated evapotranspiration surpasses a plant’s water uptake capacity, triggering cellular dehydration and oxidative stress. Inconsistent wind patterns can also compromise floral structure and hinder anemophilous pollination, reducing reproductive efficiency and crop yield (Das and Biswas, 2022). Prolonged wind exposure may induce thigmomorphogenic responses, including reduced internode length and altered distribution of biomass. Conversely, minimal wind activity especially in enclosed agricultural environments such as greenhouses limits convective air movement, thereby impairing thermal regulation and gas exchange. This, in turn, promotes microenvironments with high humidity and depleted CO_2_ levels, and such conditions encourage fungal pathogen outbreaks and suppress photosynthetic efficiency. For the aforementioned reasons, effective wind management, such as the use of shelterbelts or engineered ventilation systems is essential to maintaining optimal growing conditions that can alleviate wind-related stress (Müller et al., 2023).

#### Airborne pollution stress

Airborne pollution is a stressor that disrupts plant physiological homeostasis, interferes with metabolic functions, and undermines overall crop productivity. Major atmospheric pollutants include tropospheric ozone (O_3_), sulfur dioxide (SO_2_), nitrogen oxides (NO_x_), and particulate matter 2.5 (PM2.5), which are predominantly taken up via stomatal pathways, initiating extensive biochemical disturbances in plant tissues. Ozone exposure enhances the generation of ROS, which compromise cellular structures by damaging lipid membranes, denaturing proteins, and fragmenting nucleic acids, thereby impairing photosynthetic capacity and accelerating leaf senescence (Jimenez-Montenegro et al., 2021). SO_2_ and NO_x_ undergo apoplastic transformations through oxidation and hydration reactions, leading to intracellular acidification and suppression of vital enzymatic processes. The accumulation of particulates on leaf surfaces obstructs light absorption, disrupts stomatal regulation, and interferes with cuticular water loss. Persistent exposure to these pollutants destabilizes the redox equilibrium, downregulates carbon assimilation, and impairs source-to-sink translocation, ultimately reducing biomass and yield (Bui et al., 2022). Additionally, long-term pollution exposure weakens Systemic Acquired Resistance (SAR), increasing vulnerability to pathogens and environmental stress. Effective pollutant mitigation is essential for preserving the stability and resilience of agroecosystems.

#### Radiation stress

Radiation-induced stress adversely affects plant physiological functions, growth, and productivity. Types of ionizing radiation such as gamma rays, X-rays, and UV light can cause significant cellular damage and interfere with essential biochemical processes. Exposure to high-energy radiation often results in DNA strand breaks and mutations that impair mitotic activity, leading to stunted growth and diminished reproductive capacity. UV-B radiation, in particular, has been observed to intensify the production of ROS, which in turn causes oxidative damage. This damage includes membrane lipid peroxidation, protein degradation, and chloroplast dysfunction (Jan et al., 2022). These effects reduce photosynthetic efficiency and disturb overall metabolic stability. Radiation also affects membrane integrity and disturbs hormonal signaling pathways, further complicating plant development and morphological patterns. Sensitive plant species may exhibit symptoms such as chlorosis, necrosis, or irregular growth forms. Findings from different research groups suggest that although low radiation levels can sometimes activate stress adaptation mechanisms. However, prolonged or intense radiation exposure that surpasses the inherent protective capacity of plants can lead to significant physiological damage. To counteract these adverse effects, strategic interventions such as the use of UV-blocking films and the development of radiation-resistant crop varieties through breeding are crucial for enhancing crop resilience under radiation-induced stress (Katiyar et al., 2022).

#### Mechanical stress

The mechanical stress arising from the use of agricultural machinery and its related activities has the capacity to sometimes negatively impact plant structural integrity, physiological function, and soil quality. Ultimately, these factors contribute to compromising crop yield. It is a well-researched fact that the continual use of heavy equipment related to agricultural activities, such as tractors, plows, and harvesters, can result in the significant compaction of loose soil, reducing the microporosity that in turn reduces water infiltration and hinders proper oxygen availability to roots (Kouhen et al., 2023). These alterations hinder root respiration, restrict elongation, and impair the uptake of essential water and nutrients. Moreover, direct mechanical interference with plant organs during field operations often inflicts physical damage, including stem bending, leaf abrasion, and detachment of reproductive structures. The occurrence of these injuries reduces the effective photosynthetic surface and, in turn, disrupts assimilate allocation, ultimately risking the plant’s ability to produce a good yield. In addition, mechanical trauma can activate stress response pathways, notably through increased ethylene synthesis and ROS accumulation, which can trigger premature aging and abnormal growth responses (Cho and Nam, 2024). To alleviate these effects, practices such as Controlled Traffic Farming (CTF), low-impact machinery, and precision mechanization are vital for preserving soil structure and mitigating mechanical stress.

#### Radio frequency stress

With the invention and frequent use of 5G and 6G communication technologies in almost all areas of life to achieve the Internet of Things (IoT) and the Industrial Internet of Things (IIoT). Where Radio-frequency (RF) and Electromagnetic (EM) waves are used as sources of communication. These communication sources are within the non-ionizing spectrum of 30 kHz to 300 GHz. However, these non-ionizing ranges are now increasingly acknowledged by the scientific community as a critical stressor that influences both plant physiological homeostasis and molecular regulation. Ongoing exposure to RF sources including mobile networks, wireless communication infrastructure, and telemetry systems, has been correlated with subtle yet detrimental effects on plant biology (Kaur et al., 2021). These effects encompass alterations in membrane properties, including changes in fluidity, ion channel modulation, and disruptions to membrane potential gradients. Additionally, it is observed that the use of RF hinders the functions of crops and plant cells and their viability. This is because RF can activate oxidative stress, which is driven by the excessive generation of ROS. ROS is responsible for cell damage, lipid peroxidation, protein destabilization, and DNA fragmentation. According to findings from different research groups, reductions in seed germination rates, photosystem impairment, and decreased biomass accumulation have been highlighted. Hormonal signaling pathways, particularly those involving ABA and auxins, may also be dysregulated. Phenotypically, plants may exhibit reduced leaf area, shortened internodes, and altered chlorophyll content (Halgamuge, 2017; Tran et al., 2023).

### Biotic stress

Biotic stress in crops and plants is caused by harmful interactions with living organisms such as bacteria, fungi, viruses, insects, nematodes, and weeds. These pests and pathogens hinder plant growth, resulting in lower yields and compromised crop quality. Gaining insights into biotic stress is crucial for formulating effective pest and disease control measures. As global food security becomes increasingly critical, advancing research on resistance traits and integrated management approaches is increasingly vital to agriculture. In the coming subsection readers will find a detailed discussion of the types of biotic stresses.

#### Pathogen stress

Pathogen stress arising from fungi, bacteria, viruses, and nematodes represents a major biotic constraint that disrupts plant physiological processes, compromises cellular stability, and leads to significant yield losses. Fungal species such as Fusarium spp, Botrytis cinerea, and Phytophthora infestans infiltrate plant tissues by enzymatically degrading protective barriers such as the cuticle and cell wall, causing vascular occlusion, tissue necrosis, and impaired photosynthetic efficiency (Gorshkov and Tsers, 2022). Bacterial pathogens, including Xanthomonas and Pseudomonas spp, gain access through natural openings in plants or through physical damage, releasing effectors and cell wall-degrading enzymes that compromise membrane integrity and trigger hypersensitive responses, manifesting as chlorosis, wilting, and cell lysis. Viral pathogens, often transmitted by phloem-feeding insects, hijack host translational mechanisms to proliferate, leading to widespread symptoms such as mosaic patterns, stunted development, and organ malformations. Nematodes such as Meloidogyne spp, induce root galls that disrupt water and nutrient uptake (Desaint et al., 2021). These biotic agents often act in unison, exacerbating plant stress. Effective mitigation requires an integrated approach combining molecular diagnostics, resistant genotypes, biological control, and site-specific agronomic strategies.

#### Insect- and arthropod-induced stress

Insects and arthropods represent biotic stresses that disrupt plant physiological equilibrium, damage cellular components, and markedly decrease agricultural productivity. Key herbivorous groups, including Hemiptera (e.g., aphids, whiteflies), Thysanoptera (thrips), Lepidopteran larvae (caterpillars), and Acari (mites) employ diverse feeding mechanisms such as foliar consumption, phloem and xylem sap withdrawal via stylet insertion, and tissue injury associated with oviposition (Guedes et al., 2022). Chewing insects compromise foliar architecture and impair vascular function, thereby reducing photosynthetic efficiency and structural stability. In contrast, piercing-sucking species breach the epidermal and vascular layers, disrupting nutrient and water transport and often serving as efficient vectors for viral, bacterial, and phytoplasmal infections. Arthropod saliva frequently contains bioactive effectors that interfere with plant hormonal signaling pathways, particularly Jasmonic Acid (JA), Salicylic Acid (SA), and ethylene. This results in suppressed defense mechanisms (Jafir et al., 2023). Herbivory further induces oxidative stress through the accumulation of ROS, causing lipid peroxidation, protein degradation, and loss of membrane integrity. Symptoms include chlorosis, necrosis, impaired growth, and yield decline. Robust Integrated Pest Management (IPM) strategies involving resistant cultivars, biological control agents, and targeted agrochemical interventions are essential to mitigate these effects and ensure crop resilience.

#### Weed competition stress

The crops and plants that we grow have to compete with a variety of vegetation that is considered parasitic in nature. It can be easily observed on a daily basis that these parasitic plants have evolved with the passage of time in such a way. Now they are much better adapted to various stressors and have an exceptional ability to create vigorous biomass and reproduce copiously. Thus, the plants and crops that we cultivate to fulfill our food requirements and many other needs are under constant pressure to obtain essential resources, including PAR, soil moisture, and vital nutrients. This competitive imbalance disrupts photosynthetic carbon assimilation, limits nutrient acquisition, and constrains root development (Ronay et al., 2021). Furthermore, allelopathic weed species release chemical compounds into the rhizosphere that interfere with plant signaling, inhibit seedling establishment, and restrict root elongation through toxic effects. Dense weed infestations also modify the microenvironment by increasing relative humidity and altering canopy structure, thereby enhancing the risk of disease outbreaks and arthropod pest infestations. Collectively, these stressors can hinder the photosynthetic processes, delay developmental stages, and ultimately lead to significantly reduced crop yields (Horvath et al., 2023). Addressing these impacts requires Integrated Weed Management (IWM), which includes the strategic use of herbicides, crop diversification, selection of competitive cultivars, and site-specific agronomic interventions to sustain agroecosystem functionality and optimize resource efficiency.

#### Parasitic plant stress

Parasitic plants such as Striga and Cuscuta utilize highly specialized biotrophic mechanisms that impose a substantial physiological and metabolic burden on their host plants. Striga species infiltrate the host root cortex and form haustorial interfaces with xylem vessels that enable the extraction of water, essential minerals, and photoassimilates. This parasitic interaction disrupts transpiration dynamics and ionic equilibrium, and interferes with the translocation of carbon metabolites within the plant system (Zagorchev et al., 2021). Striga infestation leads to hormonal dysregulation, particularly in ABA signaling and strigolactone synthesis. This is accompanied by a decline in photosynthetic performance due to chlorophyll degradation and downregulation of Ribulose-1,5 bisphosphate carboxylase/oxygenase (Rubisco) activity. Cuscuta species, which lack functional roots and chlorophyll, attach to aboveground organs that tap into both the xylem and the phloem, disrupting source-to-sink relationships and altering hormonal networks, especially those involving auxins and cytokinins. Both parasitic genera provoke oxidative stress, which is characterized by increased ROS, membrane lipid peroxidation, and loss of cellular stability (Albanova et al., 2023). Control strategies include integrated management approaches involving resistance genes, pre-emergence herbicidal treatments, and modulation of rhizospheric microbial communities.

#### Animal-induced stress

Stress arising from interactions with animals including rodents, birds, livestock, and wild herbivores constitutes a major biotic constraint on the sustainability of agroecosystems and crop yield potential. Rodents cause significant damage both below and above ground by consuming seeds and vegetative organs, leading to reduced germination and impaired early plant growth (Kuka et al., 2022). Granivorous birds commonly feed on reproductive structures, resulting in grain loss and mechanical injury to inflorescences, thereby disrupting assimilate partitioning and impairing source-to-sink dynamics. Unregulated livestock access also causes extensive physical damage through trampling, leaf removal, and root exposure, which adversely affects photosynthetic capacity, turgor pressure regulation, and plant recovery potential. Wild animals and various primates inflict additional stress by stripping foliage, abrading bark, and feeding on fruits, intensifying physiological disruption (LaMalfa et al., 2021). These injuries increase ROS levels and interfere with hormone signaling, resulting in increased vulnerability to pathogen infections. Effective management requires integrated strategies involving exclusion structures, deterrents, and ecological landscape design.

#### Human-induced stress

Anthropogenic stress arising from unsustainable agricultural practices such as overexploitation of resources, improper handling of crops, monocultural farming, and suboptimal landscape configuration severely hampers plants’ physiological processes and threatens the sustainability of agroecosystems. Overharvesting interrupts developmental progression by removing vital vegetative and reproductive tissues, thereby limiting meristem function and regeneration. Mishandling during sowing, transplantation, or harvesting inflicts physical trauma, disrupts vascular continuity, and increases vulnerability to pathogenic infiltration (Demirdogen et al., 2023). Monoculture systems, with minimal genetic variability and repetitive cropping, lead to rapid soil nutrient depletion, destabilization of rhizosphere microbial communities, and a heightened risk of pest and pathogen epidemics. Additionally, poorly designed landscapes, characterized by inadequate spacing, inefficient irrigation, and a lack of crop rotation, intensify interspecific competition for essential resources such as PAR, water, and nutrients (Hernández-Ochoa et al., 2022). Collectively, these stressors impair hormonal signaling pathways, reduce photosynthetic capacity, and disrupt source-to-sink dynamics, leading to decreased biomass accumulation and diminished yield. Implementing adaptive and ecologically sound management strategies is critical to counteracting these compounded stresses and is also helpful in enhancing resilience.

## Stress indicators

**Figure 3:**
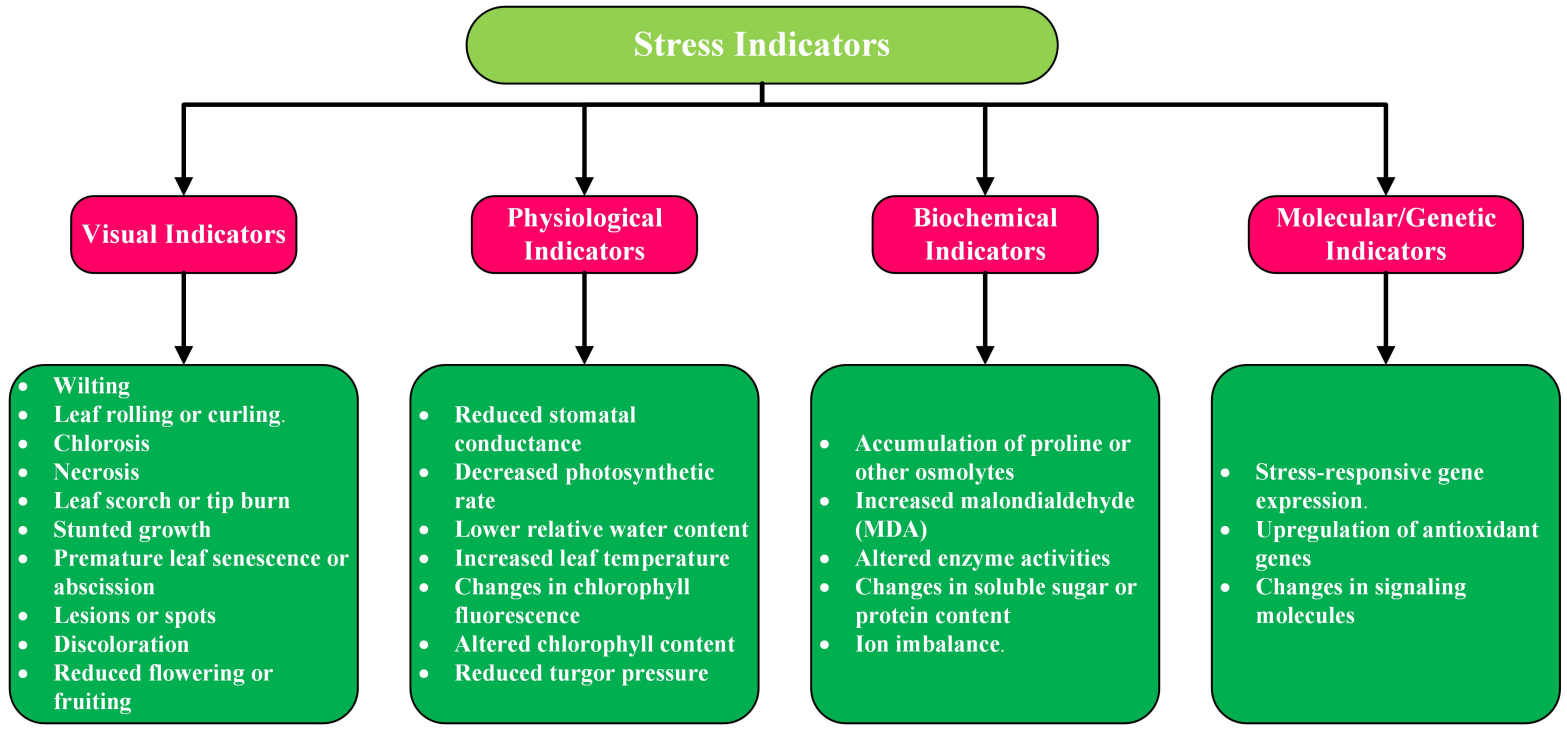
Categorization and subcategorization of stress indicators in crops and plants.

Stress indicators in plants and crops are identifiable features that show alterations in physiological, biochemical, molecular or visual traits. They assist in detecting stress caused by changes in several different variables such as water, nutrients, temperature, or diseases, allowing for prompt actions in a timely manner. Recognizing these signals is vital for improving crop health, yield, and adaptability. In this section we will briefly discuss the types of stress indicators. **Figure 3** provides their categorization and subcategorization.

### Visual indicators

Recognizable visual indicators serve as essential phenotypic indicators for assessing plant and crop stress that is triggered by abiotic or biotic factors. Where (i) wilting often reflects reduced water uptake, commonly resulting from drought, root injury, or obstruction in vascular transport. (ii) Leaf curling or rolling typically acts as a moisture conservation mechanism in response to heat or drought stress, although it may also be a reaction to chemical exposure. (iii) Chlorosis, which is visible as leaf yellowing due to chlorophyll breakdown, usually indicates deficiencies in vital micronutrients such as iron, magnesium, or nitrogen, compromised photosynthesis, or poor root zone aeration (Moustaka and Moustakas, 2023). (iv) Necrosis, which is the formation of dead tissue, frequently results from pathogenic infections, toxic chemical contact, or extreme environmental conditions. (v) Leaf scorch or tip burn is generally linked to elevated salt levels or nutrient deficiencies, especially of potassium or calcium, and may also be due to osmotic stress. (vi) Persistent stress may manifest as stunted growth, which is often tied to hormonal disruptions, nutrient scarcity, or chronic disease pressure. (vii) Premature leaf senescence or abscission is a survival response, often mediated by stress-related hormones such as ABA or ethylene. (viii) Lesions or necrotic spots are a typical visual sign of pathogen intrusion, with unique patterns aiding in pathogen identification (Berger et al., 2022). (ix) General discoloration may signal chemical contamination or systemic disease, while (x) decreased flowering or fruiting reflects impaired reproductive function due to unfavorable environmental or physiological conditions. These traits are valuable for timely diagnosis and precise management.

### Physiological indicators

Physiological indicators offer dependable and early indications of stress in plants and crops, often manifesting before any external symptoms become apparent. (i) A primary reaction to drought or elevated temperatures is a notable decrease in stomatal conductance, which typically results from a decreased turgor pressure in the guard cells. This decrease in pressure ultimately limits transpirational cooling and also curtails CO_2_ diffusion into the mesophyll, which in turn negatively impacts gas exchange efficiency (Schönbeck et al., 2023). (ii) Consequently, the net rate of photosynthesis declines due to both stomatal limitations and internal factors such as photoinhibition and Rubisco enzyme suppression. (iii) Low Relative Water Content (RWC), a key indicator of tissue hydration, significantly drops during water stress, disrupting cellular function and metabolic integrity. (iv) A rise in leaf temperature, observable via thermal imaging, signals diminished evaporative cooling caused by stomatal closure and indicates heat accumulation in foliage. (v) Alterations in chlorophyll fluorescence particularly decreases in the maximum quantum yield of photosystem that reflect impaired photochemical performance and electron transport chain instability within thylakoid membranes (Soltabayeva et al., 2021). (vi) Shifts in chlorophyll content, as measured by Soil Plant Analysis Development (SPAD) indices or spectrophotometric analysis, denote pigment degradation and reduced efficiency in light energy absorption. (vii) Furthermore, a decline in turgor pressure inhibits cell expansion and structural support, which directly influences growth and tissue resilience. Monitoring these metrics facilitates the accurate, real-time evaluation of plant stress responses and informs targeted mitigation strategies.

### Biochemical indicators

Biochemical markers are highly accurate and responsive tools for the early identification of stress in plants, often manifesting before any discernible physiological or structural alterations may occur. (i) A central adaptive response to abiotic stress involves the increased biosynthesis and accumulation of compatible osmolytes such as proline and glycine betaine, along with other low-molecular-weight protective compounds. These molecules contribute to maintaining osmotic stability, safeguarding proteins and cellular integrity, and detoxifying ROS under adverse conditions such as drought, salinity, and heat stress (Iqbal et al., 2021). (ii) The buildup of Malondialdehyde (MDA), a lipid peroxidation product derived from polyunsaturated fatty acids, serves as a reliable indicator of oxidative membrane injury. (iii) Stress conditions also modulate the activity of major antioxidant enzymes, including SOD, CAT, and Various Peroxidases (POD, APX), reflecting an activated defense network aimed at regulating ROS levels and sustaining redox balance. (iv) Additionally, fluctuations in soluble sugar and protein concentrations highlight reprogrammed metabolic activity that supports osmoprotection, energy distribution, and cellular restoration. (v) Disruptions in ion homeostasis particularly in Na^+^, K^+^, and Ca^2+^ gradients further characterize ionic and osmotic stress, influencing membrane dynamics, enzyme functions, and intracellular signaling (Ben et al., 2023). Altogether, these biochemical alterations provide precise, quantifiable insights into plant stress physiology and are critical for advanced phenotyping, stress diagnostics, and precision agriculture interventions.

### Molecular/genetic indicators

Molecular and genetic indicators provide a highly specific and flexible means for the early identification of plant responses to various stresses, operating across transcriptional, post-transcriptional and signaling networks. (i) A fundamental aspect of the plant’s molecular response involves the selective expression of key stress-related genes, including Dehydration Responsive Element Binding proteins (DREBs), HSPs, and Response-to-Dehydration 29A (RD29A) (Datir and Regan, 2022). These genes are central components of sophisticated regulatory circuits that govern stress detection and adaptation. DREB proteins regulate gene expression in response to drought and salinity by binding to Dehydration-Responsive Element/C-Repeat (DRE/CRT) motifs within promoter regions, while HSPs function as molecular chaperones that preserve protein integrity under heat and oxidative stress. RD29A is a well-established genetic marker for water deficit and osmotic stress that responds to ABA-mediated and independent pathways. (ii) In response to abiotic stress, there is also an upregulation of gene coding for antioxidant enzymes such as SOD, CAT, and APX, which collectively detoxify ROS and maintain redox homeostasis. (iii) Concurrently, fluctuations in signaling molecules such as ABA and ethylene initiate stress-related signaling that regulates gene expression and physiological responses (Gowayed and Abd El-Moneim, 2021). Monitoring these molecular signatures enables precise diagnostics and supports advanced molecular breeding and genetic engineering efforts to enhance stress tolerance in crops. In **Table 2**, we present a summarized overview of the various categories of stress indicators to facilitate a better understanding for readers. The purpose of this table is to consolidate the relevant information regarding each type of indicator whether visual, physiological, biochemical, or molecular/genetic, into a single, organized format. By doing so, readers can conveniently access this collective knowledge along with concise explanations, allowing for quicker interpretation and improved clarity on the aforementioned concepts discussed in this section.

**Table 2:** A List of stress indicators with their concise explanation.

S. no	Categories	Stress indicators	Description/Examples	References
1	Visual	Leaf discoloration, wilting, necrosis, stunted growth, chlorosis, leaf curling	Observable symptoms caused by drought, nutrient deficiency, pest attacks, or disease	(Moustakas et al., 2022; Stutsel et al., 2021)
2	Physiological	Stomatal conductance, photosynthetic rate abnormality, transpiration rate abnormality	Reflects internal plant functioning, changes in gas exchange or water retention during stress	(Kimm et al., 2021; Wen et al., 2023)
3	Biochemical	Proline content, MDA, ROS, antioxidant enzymes SOD, CAT, POD	Indicators of oxidative stress and biochemical defense mechanisms	(Iqbal et al., 2021; Abdelaal et al., 2022)
4	Molecular/Genetic	Gene expression (e.g., HSPs, stress-responsive transcription factors), DNA methylation patterns	Reveals the stress response at the genetic level, including the activation of stress-regulated genes or epigenetic changes	(Gowayed and Abd El-Moneim, 2021; Ghonaim et al., 2023)

## Stress assessment methodologies

**Figure 4:**
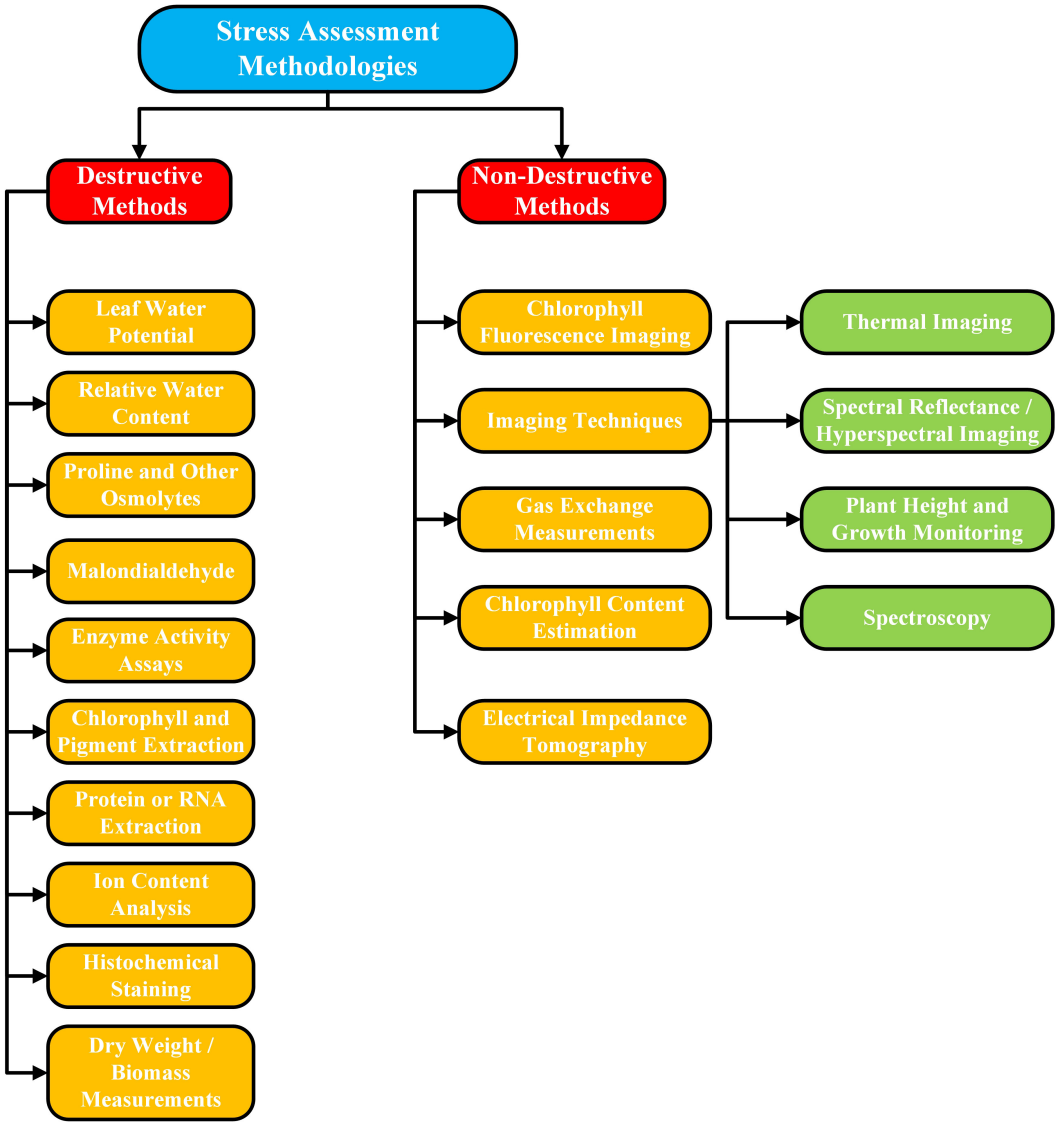
A flow diagram elaborating the categorization and subcategorization of stress assessment destructive and non-destructive methodologies.

Crop stress evaluation is performed using both destructive and non-destructive approaches to assess the physiological, biochemical, and structural changes that occur in plants under biotic or abiotic stress. Traditional destructive methodologies, once widely used by researchers and practitioners, require the collection of crops and plant samples for detailed laboratory analysis. In comparison to non-destructive methods, including thermal imaging, the use of chlorophyll sensors, and spectral tools, it allows for monitoring without damaging the health or yield of the plant. This section presents an in-depth discussion of these destructive and non-destructive approaches. To enhance the readers’ comprehension in a clearer understanding of the content, we have included a flow diagram in **Figure 4**. This is designed in a structured manner so that all the various stress assessment methodologies discussed in the same section are presented in categories. The intent behind incorporating this visual representation is to assist readers in grasping the relationships and distinctions among the different approaches more effectively, by offering a summary of the content at a glance.

### Destructive methods

Destructive techniques for evaluating plant stress include the collection and laboratory analysis of plant tissues using methods such as biochemical testing and histological examinations. While these methods yield precise internal information, they necessitate the removal of plant parts or entire plants, resulting in the sampled tissue or plant dying post-analysis.

#### Leaf water potential (pressure chamber method)

**Figure 5:**
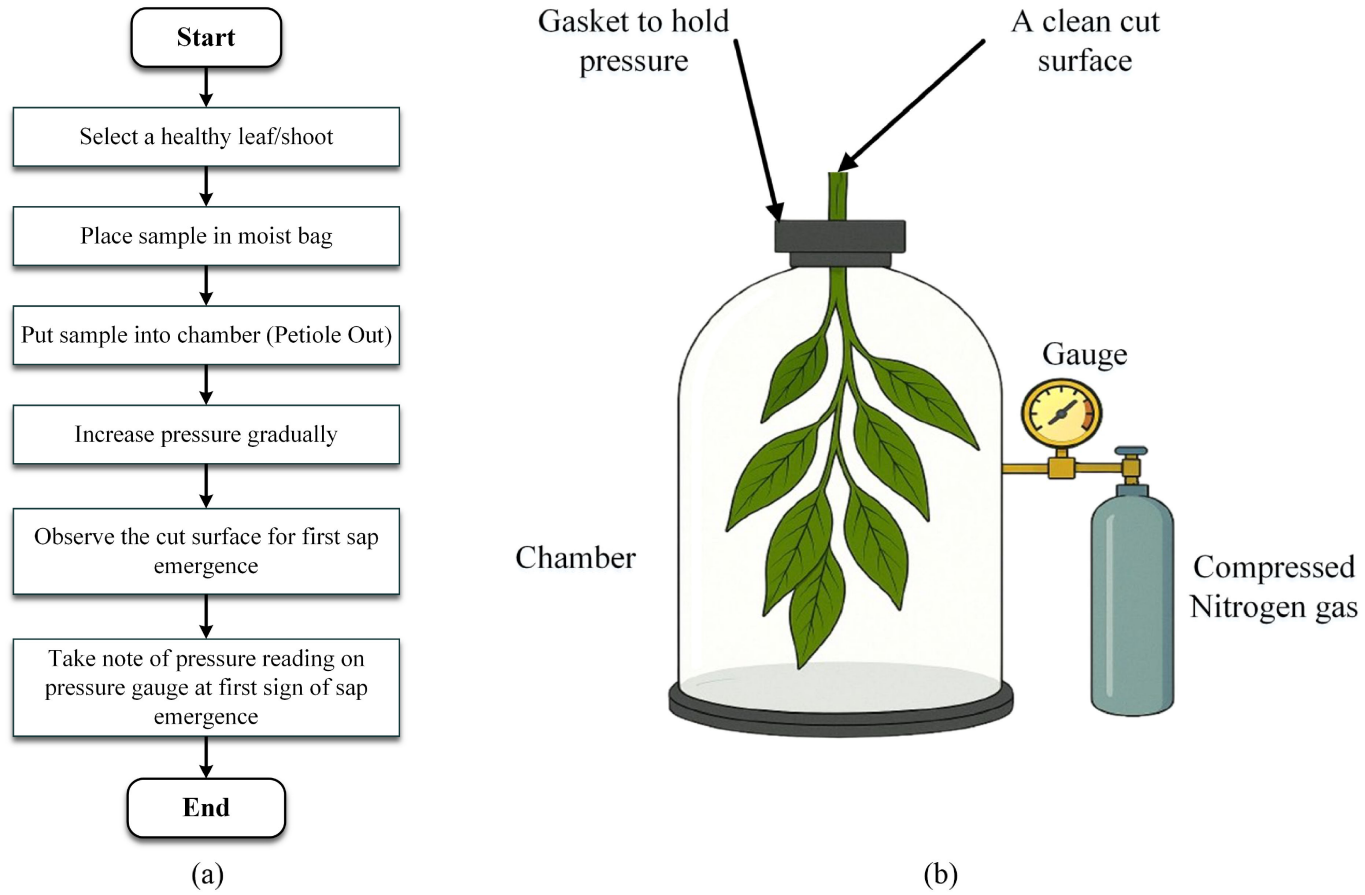
**(a)** A basic flow diagram of the leaf water potential process through a pressure chamber method and **(b)**. A very basic apparatus setup to understand the process (Awad-Allah, 2020).

Leaf water potential (Ψ_leaf) quantifies the total potential energy of water within leaf tissues and is a key determinant of water movement through the plant’s hydraulic continuum. The Pressure Chamber Method, a widely recognized destructive approach, provides accurate measurements of Ψ_leaf in both field and experimental settings. The method involves excising a mature, physiologically active leaf or shoot, ideally during midday when water stress is typically at its peak and securing it in an airtight pressure chamber, with the severed end extending through a tightly sealed gasket (Rodriguez-Dominguez et al., 2022). Compressed nitrogen is gradually introduced to increase the chamber’s pressure, effectively countering the negative pressure (tension) in the xylem. When the sap emerges at the cut surface, the internal tension is balanced by the external pressure, which is recorded in megapascals (MPa) and that reading corresponds to the leaf’s water potential. More negative values of Ψ_leaf indicate higher levels of water stress and reduced water availability to the plant. This method yields high-resolution quantitative data that are critical for optimizing irrigation schedules, assessing drought resilience, and modeling plant physiological responses to environmental stress. The formula to calculate leaf water potential is Ψ_Leaf = Ψ*_s_* + Ψ*_p_* where Ψ*_s_* is the solute potential and Ψ*_p_* is the pressure potential (Ding et al., 2021). A simplified process flow is illustrated in **Figure 5a**, while a basic setup of the equipment to perform the aforementioned task is shown in **Figure 5b**.

#### Relative water content

RWC is an important physiological metric for assessing the water status of plant tissues under various environmental conditions, particularly drought. RWC reliably reflects the extent of water retention within plant cells and indicates the plant’s ability to maintain turgor pressure, which is crucial for sustaining metabolic activities and supporting cell expansion during stress (Rashid et al., 2022). The assessment of RWC involves three main stages: (i) the Sampling Phase, in which mature, healthy leaves are harvested from a consistent nodal position across plants to ensure uniformity. Immediately after collection, the Fresh Weight (FW) is recorded to capture the leaf’s current water content. (ii) the rehydration phase, during which the samples are immersed in deionized water and kept in darkness at room temperature (approximately 22°C to 25°C) for 4 to 6 hours, allowing the leaves to fully rehydrate and reach maximum turgor. After rehydration, surface moisture is gently removed, and the Turgid Weight (TW) is measured. (iii) the drying phase, during which the leaves are dried in an oven at 70°C for 24 to 48 hours to achieve a constant weight, known as the Dry Weight (DW), which represents their solid biomass (Ilyas et al., 2021). The formula to calculate the RWC is RWC (%) = [(FW − DW)/(TW − DW)] × 100. This calculation provides an accurate measure of tissue water deficit. High RWC values are associated with better water retention and stress tolerance, whereas low values indicate dehydration and increased vulnerability. RWC is widely used in ecological and physiological research and plays a vital role in breeding programs focused on improving stress resistance.

#### Quantification of proline and other osmolytes

Quantifying proline and other compatible osmolytes is a pivotal technique used for analyzing plant physiological responses to stress conditions such as drought, high salinity, and temperature fluctuations. These adverse conditions disrupt cellular homeostasis, triggering the upregulation and accumulation of low-molecular-weight osmolytes such as proline, glycine betaine, and soluble sugars. That plays an essential role in osmotic adjustment, and membrane and protein stabilization, with the mitigation of oxidative stress through ROS scavenging (Spormann et al., 2023). The standard procedure for proline determination involves homogenizing fresh leaf tissue in 3% sulfosalicylic acid, followed by centrifugation to obtain a clear supernatant. This extract is then reacted with an acid ninhydrin solution (prepared using 1.25 g ninhydrin in a mixture of glacial acetic acid and phosphoric acid) and incubated at 100°C for 60 minutes to facilitate chromophore formation. Once cooled, the mixture is extracted with toluene, and the absorbance of the resulting colored complex is measured using a UV spectrophotometer. The concentration of proline can then be observed in comparison to the calibration curve of L-proline standards (Zdunek-Zastocka et al., 2021). However, researchers have also mentioned that other types of related osmolytes, that are equally important, can be analyzed by using distinct, specific colorimetric assays, which consist of the phenol sulfuric acid method for sugar evaluation, and also incorporate more progressive state-of-the-art techniques into existing approaches, such as High Performance Liquid Chromatography (HPLC). We can conclude that the described analytical approach is crucial for understanding stress tolerance mechanisms and is also helpful in identifying stress-resilient genotypes in crop improvement programs.

#### Malondialdehyde and lipid peroxidation assay

The MDA and Lipid Peroxidation (LPO) assay is a vital biochemical method for determining the extent of oxidative damage to plant cell membranes caused by stress, especially under conditions such as drought, salinity, and extreme temperatures. LPO is a key marker of oxidative stress, triggered by ROS attacking polyunsaturated fatty acids in membrane structures. This leads to the formation of MDA, a reactive aldehyde and widely accepted biomarker of oxidative damage (Shahid et al., 2022). MDA quantification is most commonly carried out using the Thiobarbituric Acid Reactive Substances (TBARS) assay. In this protocol, the users will homogenize the tissues from a fresh plant; the part of the plant that can be used is the leaves. In this process the leaves are homogenized in 0.1% to 1% trichloroacetic acid (TCA). This homogenization helps in the precipitation of proteins, which makes extracting soluble metabolites possible. After centrifugation, the supernatant is mixed with thiobarbituric acid (TBA) and dissolved in 20% TCA. The reaction mixture is incubated at 95°C for 30 to 60 minutes, which helps in promoting the formation of a pink-colored MDA-TBA complex. After cooling, absorbance is measured at 532 nm using a spectrophotometer, with a correction at 600 nm to account for background interference (Wang et al., 2022). MDA content is then determined with the help of either a standard curve or by using a molar extinction coefficient. This assay is essential for investigating lipid peroxidation and screening plant genotypes for oxidative stress resilience.

#### Enzyme activity assays

Antioxidant enzyme activity assays, specifically targeting SOD, CAT, and POD, are essential biochemical methods for characterizing plant responses to oxidative stress triggered by stress conditions such as drought, salinity, and temperature extremes. These enzymes play a key role in the enzymatic antioxidant defense mechanism by neutralizing ROS generated during stress-induced metabolic dysfunction (Kavian et al., 2022). Typically, in this process, plant tissues, most commonly leaves, are homogenized in an ice-cold extraction medium, such as potassium phosphate buffer (pH 7.0), supplemented with protective additives such as Ethylenediaminetetraacetic Acid (EDTA) and 1% Polyvinylpyrrolidone (PVP) to inhibit phenolic interference and preserve enzymatic integrity. The homogenate is then centrifuged at 12,000 to 15,000 × g for 15 to 20 minutes at 4°C to collect a clear enzyme-containing supernatant. SOD activity is measured by its ability to inhibit the photoreduction of nitroblue tetrazolium (NBT), with absorbance monitored at 560 nm. CAT activity is evaluated by tracking the reduction in absorbance at 240 nm as hydrogen peroxide is decomposed enzymatically. POD activity is quantified by observing the oxidation of guaiacol in the presence of hydrogen peroxide, and measuring it at 470 nm (Osei et al., 2022). These spectrophotometric evaluations offer precise indicators of a plant’s oxidative defense status and are indispensable in stress physiology and genotype selection programs.

#### Chlorophyll and pigment extraction

It has been observed that whenever a plant or a crop is affected by a factor that has the ability to induce stress in vegetation, such as drought, salinity, intense light exposure, and extreme temperatures, it may be necessary to extract the chlorophyll and pigments for analysis to evaluate the efficiency of the photosynthetic process in plants. This allows for determining whether the plant is performing optimally and whether a good yield can still be obtained or if some kind of countermeasures are required to put the crop or the plant back on track to achieve a sufficient yield. Plants under environmental stress have disrupted pigment biosynthesis and accelerated chlorophyll degradation. This hinders the ability of the plant to use the captured light and perform the process of photosynthesis efficiently. Quantitative analysis of chlorophyll a, chlorophyll b, and auxiliary pigments such as carotenoids provides a dependable indicator of photosynthetic competence and stress-induced deterioration (Berhe et al., 2024). The standard method involves grinding freshly collected leaf tissues in an appropriate organic solvent, which is commonly 80% acetone, absolute ethanol, or Dimethyl Sulfoxide (DMSO). This process is done under dim light to avoid pigment degradation. The extract is then centrifuged at 10,000 to 15,000 × g for 10 to 15 minutes to yield a pigment-rich supernatant, which is used for spectrophotometric assessment. The users will then take absorbance readings at specific wavelengths of 663 nm for chlorophyll a, 645 nm for chlorophyll b, and 470 nm for carotenoids. Then, the pigment concentrations can be determined by using standardized equations, such as those developed by Arnon or Lichtenthaler. Once the calculations are obtained, the results are analyzed and expressed in relation to the fresh weight of the plant tissue (Saini et al., 2022).A reduction in pigment levels signifies oxidative damage, disruption of chloroplast function, and metabolic imbalance, underscoring the value of this assay in stress diagnostics and crop improvement programs.

#### Protein or RNA extraction

Protein and Ribonucleic Acid (RNA) extraction are fundamental molecular techniques that are very crucial for characterizing the functional responses of plants to stressors at the transcriptomic and proteomic levels. RNA is commonly extracted using phenol-chloroform-based methods such as the Total RNA Isolation Reagent (TRIzol) or commercial silica-based spin column kits. To ensure high-quality RNA that may be suitable for downstream applications, Deoxyribonuclease I (DNase I) treatment is applied to remove any contaminating genomic DNA. Quantitative Real-Time Polymerase Chain Reaction (qPCR) is utilized to evaluate the expression levels of critical stress-responsive genes in plants. These include genes that encode antioxidant enzymes such as SOD and CAT, HSPs, and transcription factors such as DREB and WRKY (named after the SPF1 protein cloned from sweet potato). All of these play vital roles in defending against environmental stresses such as drought, salinity, cold, and pathogen invasion (Khurshid et al., 2021). To get a better view of gene expression, RNA sequencing offers high-throughput analysis that has the capability of detecting genome-wide transcriptomic changes, which in turn makes it possible to reveal the regulatory networks associated with stress adaptation. Recovery of stress-inducible proteins is only possible under denaturing or native conditions. These proteins are subsequently characterized using electrophoretic techniques such as Sodium Dodecyl Sulfate Polyacrylamide Gel Electrophoresis (SDS-PAGE), immunodetection via western blotting, or more sophisticated proteomic approaches including Liquid Chromatography Tandem Mass Spectrometry (LC-MS/MS) (Wang et al., 2024). As discussed, these analyses can expose crucial information about the post-translational modification, enzyme activity modulation, and signal transduction pathways that are triggered by environmental stimuli. The accuracy and reliability of both RNA- and protein-based investigations depend on rigorously optimized extraction protocols that preserve biomolecule integrity, inhibit enzymatic degradation, and avoid contamination. Thus, it can be concluded that, taken together, transcriptomic and proteomic methodologies can provide a comprehensive molecular framework. This framework can be used for the understanding of plant stress responses, which can in turn play a pivotal role in guiding the development of genetically enhanced, stress-tolerant crop varieties.

#### Ion content analysis

Ion content analysis, notably the precise measurement of Na^+^ and K^+^ levels, is an essential physiological technique for assessing how plants respond to and adapt to saline stress conditions. This is because an immense amount of salinity in the soil will normally result in higher Na^+^ content in plant tissues. This, in turn, damages the normal ionic balance of a crop/plant, and prohibits K^+^ uptake. If these abnormalities occur due to severe salinity, they will hinder key cellular functions including enzymatic activity, protein synthesis, and membrane potential regulation (Kumar et al., 2021). For analysis, plant tissues such as leaves, stems, or roots are carefully collected and thoroughly washed to remove surface salts, oven-dried to achieve constant weight, and finely pulverized. The powdered material undergoes acid digestion using concentrated nitric acid or a nitric-perchloric acid mixture under controlled conditions to extract ionic constituents. These digests are then appropriately diluted and analyzed using techniques such as flame photometry, Atomic Absorption Spectrophotometry (AAS), or Inductively Coupled Plasma Optical Emission Spectrometry (ICP-OES) for precise ion quantification (Singhal and Singh, 2024). Based on the preceding discussion on salinity, it can be concluded that the Na^+^/K^+^ ratio in the examined soil serves as a reliable physiological marker for assessing salinity tolerance. A lower Na^+^/K^+^ ratio indicates effective ion selectivity, efficient osmotic regulation, and the preservation of cellular ion balance under saline conditions.

#### Histochemical staining

Histochemical staining is a highly specialized cytochemical technique that can be utilized for the *in situ* detection of stress-induced biochemical alterations in plant tissues. This technique provides detailed resolution at both the cellular and subcellular levels. It employs chromogenic or fluorogenic reagents to identify molecular markers related to oxidative injury, enzyme activity, or cell viability under stress conditions. Diaminobenzidine (DAB) is used to detect Hydrogen Peroxide (H_2_O_2_) by producing a localized brown precipitate, whereas NBT reacts with superoxide anions to form a blue formazan deposit. Both serve as spatial indicators of ROS presence (Yadav et al., 2021). As part of this method the users first select the appropriate plant or crop samples necessary to perform the aforementioned histochemical staining process such as leaves and roots. These parts are carefully detached and infiltrated under an artificially created vacuum in the presence of a selected staining reagent. Once this step is completed, the extracted and processed plant part will be incubated with the staining reagent in the absence of light at a precisely controlled temperature to ensure that efficient reaction kinetics are achieved. Following staining, the tissues are cleared using ethanol or acetic acid, with glycerol solution used to eliminate the chlorophyll and improve visualization (De Palma and Fett-Neto, 2024). The stained sections are then analyzed using light or fluorescence microscopy to enable a semi-quantitative evaluation of oxidative stress patterns and spatial mapping of plant defense responses.

#### Dry weight/biomass measurement

Estimating dry weight or biomass is a critical physiological technique employed to evaluate plant growth responses and assess the extent of stress-induced effects under both abiotic and biotic challenges. To perform these measurements, a specific process is used that includes obtaining plant parts such as leaves, stems, roots, and, in some site-specific cases, the entire plant. Once these samples are collected, they are carefully cleaned to prevent any kind of external debris from contaminating them. Then, the samples are gently bottled to ensure that residual moisture on their surface can be properly eliminated (Qu et al., 2021). These samples are then oven-dried at a regulated temperature, generally between 65°C and 80°C, for 48 to 72 hours or until a constant weight is achieved, ensuring complete dehydration. After drying, the samples are accurately weighed using a sensitive analytical balance to determine the dry biomass, which reflects the plant’s total organic matter excluding water content. Crops or plants may exhibit signs of impaired photosynthetic activity, altered metabolic pathways, and restricted cellular proliferation (Chandrasekaran, 2022). Once a farmer or lab analyst detects any impairment, dry weight analysis can provide a reliable and integrative metric for evaluating stress tolerance and genotypic variation in plant physiological and agronomic studies.

### Non-destructive methods

Non-destructive approaches offer a fast and reliable evaluation of plant and crop stress in real time while preserving the integrity of the samples. Methods such as chlorophyll fluorescence imaging, thermal imaging, hyperspectral analysis, and canopy reflectance measurement allow for the ongoing observation of physiological and biochemical variations, aiding in the early detection of stress and enhancing precision farming and crop management practices.

#### Chlorophyll fluorescence imaging

**Figure 6:**
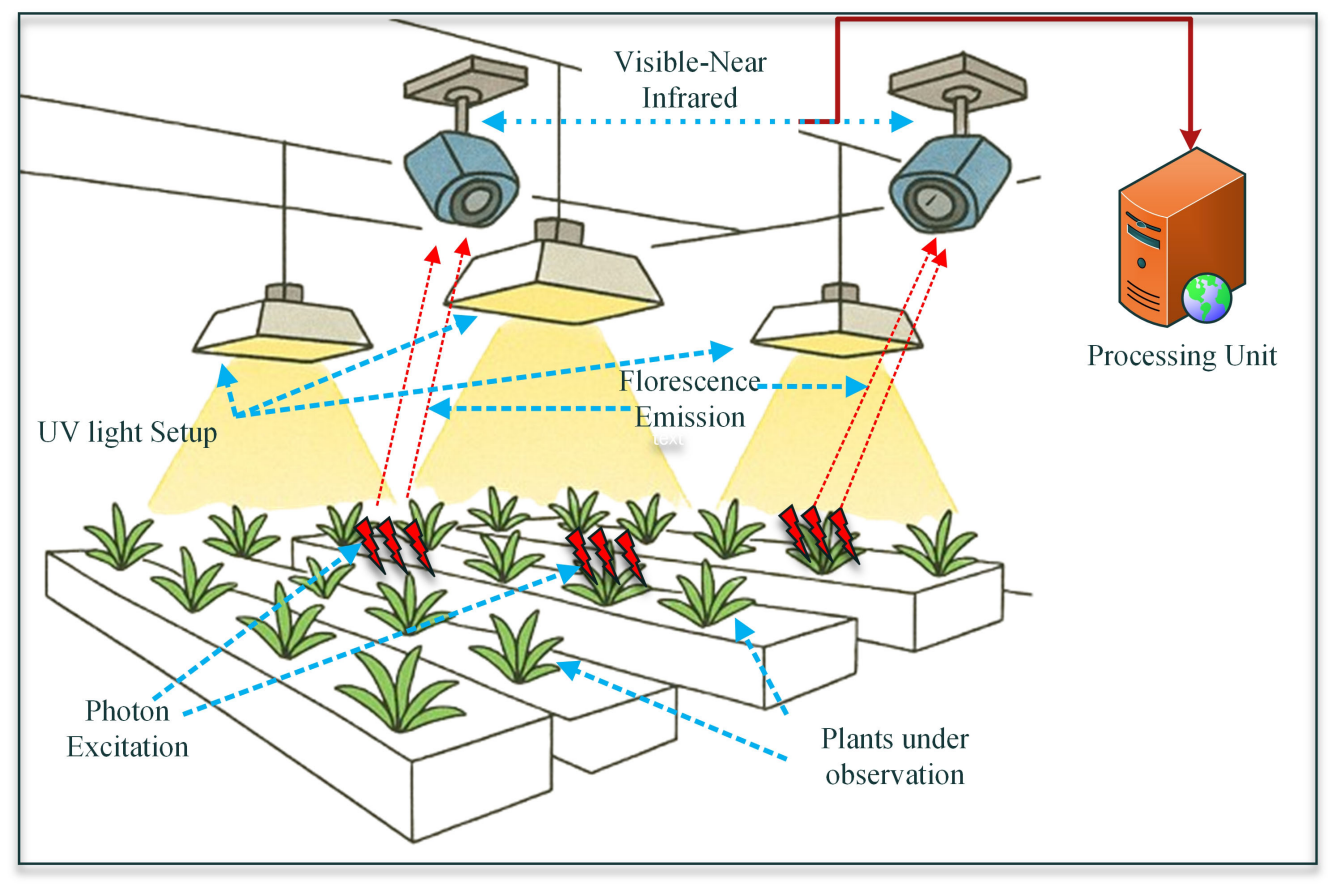
A schematic diagram showing the fundamental setup of a chlorophyll fluorescence imaging system in a closed monitoring environment (Park et al., 2024).

Chlorophyll fluorescence imaging is a precise, non-destructive method used to evaluate photosynthetic performance and detect early stress indicators in plants at a high spatial resolution. This technique is based on the idea that a small amount of light absorbed by Photosystem II (PSII) is re-emitted as fluorescence. Variations in this signal reflect the plant’s physiological state regarding photosynthesis (Moustaka and Moustakas, 2023). Under optimal conditions, the absorbed energy is largely utilized for photochemical processes. However, exposure to environmental stresses such as drought, salinity, temperature extremes, or nutrient deficiencies reduces the efficiency of electron transport in PSII, leading to increased fluorescence output. Utilizing the Pulse Amplitude Modulated (PAM) fluorometry combined with advanced imaging systems, researchers can measure parameters such as maximum quantum yield (Fv/Fm), effective quantum yield (ΦPSII), and NPQ, which reflect declines in PSII efficiency and indicate photoinhibition under stressed conditions. This approach has the capability to enable high-throughput, real-time phenotyping along with precise spatial mapping of photosynthetic stress responses. This ultimately makes it an essential tool for accessing plant physiology and stress diagnostics, bringing the users closer to achieving precision agriculture (Park et al., 2024). A formal blueprint of the explained process is provided in **Figure 6**, so that readers can better understand the schematic and implement it further if needed.

#### Imaging techniques

**Figure 7:**
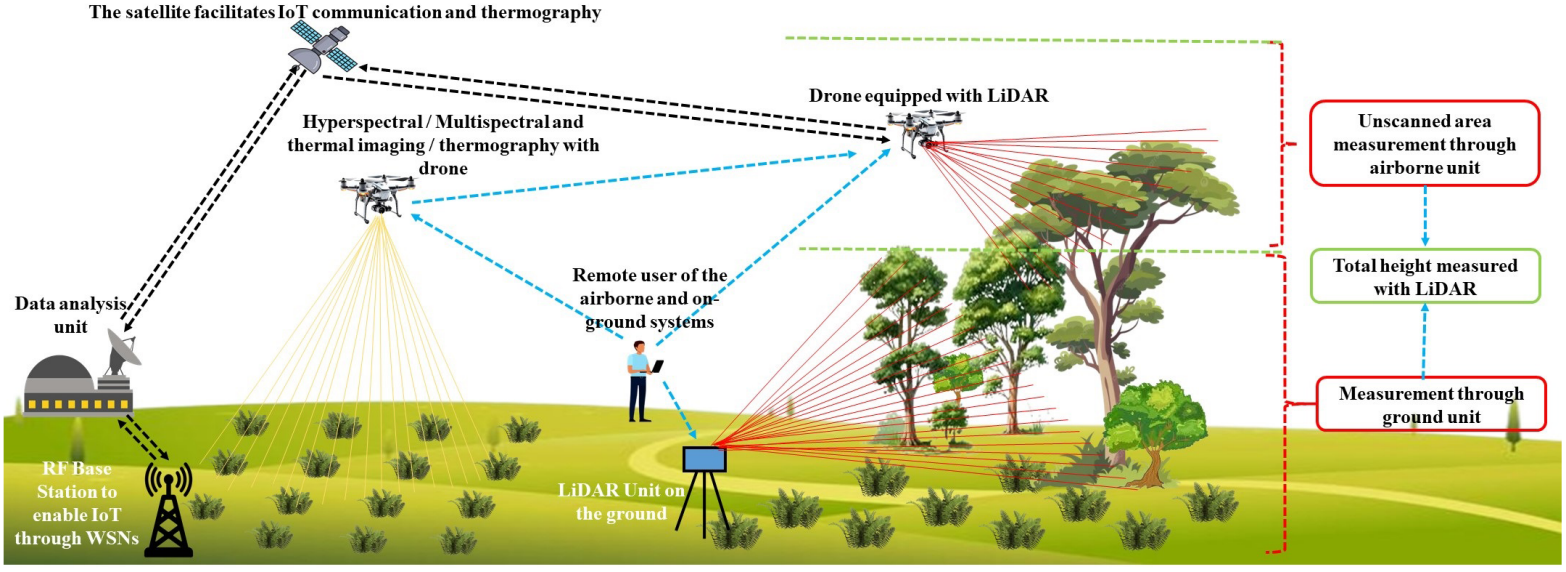
A basic schematic diagram showing the stress detection approaches using non-destructive techniques such as lidar, hyperspectral imaging, and thermography (Mielcarek et al., 2018; Ojoatre, 2016).

Imaging technologies, including Red Green and Blue (RGB) cameras, multispectral imaging, and thermal imaging (thermography), play a crucial role in identifying stress in crops and plants. Integrating these imaging systems with drones and satellites within the scope of Wireless Sensor Networks (WSNs) effectively achieves this objective, enabling comprehensive stress detection in agricultural environments. RGB imaging captures visual patterns and color variations in foliage, which often reflect nutrient deficiencies or the presence of a disease. Multispectral systems gather data across different spectral bands, enabling the assessment of plant health through indices such as the Normalized Difference Vegetation Index (NDVI). Thermal imaging, whether deployed via satellite or drone, tracks variations in canopy temperature, offering insights into heat stress or impaired transpiration resulting from drought conditions. Additionally, Light Detection and Ranging (LiDAR) provides a three-dimensional view of the canopy structure, enhancing stress assessment capabilities. Collectively, these non-invasive tools support real-time observation, early diagnosis of stress symptoms, and precision agriculture strategies, thereby improving crop health management and boosting productivity across varying environmental conditions. In the subsection of this part of the review, readers will find technical details of these non-destructive imaging stress assessment methods. **Figure 7** provides a basic layout that is aimed at stress detection using non-destructive techniques such as LiDAR, hyperspectral imaging, and thermography to complement the discussion in sections 4.2.2.1, 4.2.2.2 and 4.2.2.3.

##### Thermal imaging (infrared thermography)

Thermal imaging, also known as infrared thermography, is a technique that is utilized to monitor physiological stress in plants by identifying spatial temperature differences across the canopy. It operates on the principle that all surfaces emit infrared radiation according to their temperature, with stress-related changes in plant transpiration leading to variations in thermal emissions (Hernanda et al., 2024). Under abiotic stress conditions such as drought, salinity, or pathogen invasion, stomatal closure reduces transpiration, resulting in elevated leaf temperatures. Thermal sensors that can capture high-resolution images are used to detect thermal anomalies in the field or in the canopy of tall plants in dense vegetation. Through this detection process, the users can produce thermographic images that may offer temperature data at the pixel level. This method enables the detection of stress indicators before any visible symptoms develop, serving as a proactive tool for managing crop health. Additionally, thermal imaging is well suited for integration with UAVs and satellite platforms, supporting high-throughput phenotyping and large-scale agricultural surveillance (Pineda et al., 2020). The technology’s contactless nature ensures that plant integrity remains intact, making it particularly valuable for ongoing studies of stress responses under fluctuating environmental conditions.

##### Spectral reflectance/hyperspectral imaging

Spectral reflectance and hyperspectral imaging are advanced, non-invasive remote sensing methodologies engineered for the detailed, high-resolution detection of both biotic and abiotic stressors in agricultural systems. These techniques leverage the unique spectral reflectance patterns of vegetation across an extensive wavelength continuum, from the visible to the Near Infrared (NIR), and Shortwave Infrared (SWIR) regions (Ogawa et al., 2024). Whenever a crop is subjected to stressors such as drought, nutrient deficiency, salinity, or disease, various researchers have pointed out that critical physiological parameters such as chlorophyll content, leaf moisture status, and cellular organization undergo measurable changes. These are ultimately reflected in altered spectral signatures due to these stressors. Hyperspectral sensors acquire highly resolved spectral data across hundreds of contiguous, narrow wavelength bands, facilitating the precise detection of subtle shifts in reflectance. Using this rich spectral information, users of this technology – i.e., the farmers - can measure vegetation indices such as the NDVI, Red Edge Inflection Point (REIP), and Photochemical Reflectance Index (PRI). Once these vegetation indices-based data are collected, we can computationally derive and analyze the stress indicator to which the vegetation indices refer (Roy et al., 2023). These advanced technologies are now allowing us to detect stress early, often before there are any visible symptoms and empower us to move a step forward to achieve precision agriculture.

##### Plant height and growth monitoring

Assessment of plant height and growth using LiDAR technology provides a high-throughput, non-invasive approach to detecting structural modifications in crops resulting from abiotic and biotic stress in agricultural environments. LiDAR operates by emitting laser beams that strike plant surfaces and capture the reflected signals, generating dense 3D point clouds that offer precise characterization of canopy structure, vertical growth dynamics, and spatial variability at fine temporal and spatial resolutions (Hütt et al., 2023). Various research groups have observed that under stress conditions such as drought, salinity, nutrient deficiency, or pathogen exposure, plant growth is typically constrained, leading to reductions in elongation, biomass accumulation, and alterations in canopy configuration. The aforementioned morphological changes due to recent or ongoing stress can be measured with LiDAR technology, which can derive features that include plant height profiles, volumetric growth metrics, and canopy surface reconstructions. We have found that LiDAR systems can provide benefits in agricultural stress conditions; therefore, integrating these beneficial outputs with ML models can help users in the early identification of growth-related stress before visible symptoms appear (Rivera et al., 2023). Ultimately, it can be concluded that, as a robust and scalable technology, LiDAR can enhance the monitoring of temporal growth trends, enabling advanced phenotyping, precision agriculture, and stress response modeling.

##### Spectroscopy

Non-destructive stress detection using spectroscopic techniques is a reliable, high-resolution approach for identifying physiological changes in crops and plants without altering their physical form. Operating within spectral domains such as the visible, NIR, and hyperspectral bands, these methods analyze the interaction between incident light and plant tissues to measure their reflectance and absorption profiles. These spectral outputs offer valuable information on key biochemical and structural features, such as chlorophyll content, carotenoid presence, leaf hydration levels, and cellular architecture (Zahir et al., 2022). Abiotic and biotic stressors such as water scarcity, salinity, nutrient imbalance, and pathogen attacks cause distinct shifts in spectral signatures that can be detected before visual symptoms appear. To interpret these high-dimensional datasets effectively, advanced computational tools, including ML algorithms and multivariate statistical models, are employed to improve classification accuracy. The deployment of these systems via UAVs and ground-based platforms enables efficient, scalable, and spatially precise monitoring, making this technology a vital component of real-time crop stress diagnostics and data-driven precision agriculture (Cozzolino and Roberts, 2016). It is particularly important for research students to recognize that, while spectroscopy and hyperspectral imaging both examine the interaction of light with matter, they are inherently different in scope and application. Spectroscopy is a broad scientific discipline that encompasses various methods of analyzing light to identify and characterize materials based on their spectral properties. Hyperspectral imaging, on the other hand, is a more focused technique within the field that merges spectral analysis with imaging technologies, enabling the concurrent capture of both spectral and spatial information across an observed target or scene.

#### Gas exchange measurements

Gas exchange analysis is a vital physiological technique for accurately assessing plant responses to abiotic stress. It measures real-time gas fluxes associated with photosynthesis and transpiration. This approach employs advanced Infrared Gas Analyzers (IRGAs) paired with controlled cuvette systems to monitor essential parameters, including net CO_2_ assimilation, stomatal conductance, transpiration rate, and intercellular CO_2_ concentration. Under environmental stressors such as drought, salinity, extreme temperatures, or nutrient limitations, plants undergo shifts in stomatal regulation and metabolic activities, resulting in noticeable declines in carbon assimilation and alterations in transpiration dynamics (Pflüger et al., 2024). From the literature, we have observed that the aforementioned stresses can reduce a plant’s ability to signal a reduction in gas exchange efficiency and this specific property can be detected much earlier than any visible symptom appears. Therefore, it can be concluded that the non-destructive gas exchange profiling can have a crucial role in evaluating PSII function and determining water use efficiency. This approach offers users (farmers or field analysts) valuable insight into plant stress responses and helps them make informed decisions regarding genotype screening, agronomic strategy development, and environmental stress modeling within precision agriculture frameworks (Abdelhakim et al., 2021).

#### Chlorophyll content estimation

Chlorophyll content measurement is a widely adopted, non-destructive technique for assessing the physiological state of plants, especially when under stress. One of the most commonly utilized instruments for this purpose is the SPAD meter, which determines relative chlorophyll levels by measuring the differential transmission of red (650 nm) and NIR (940 nm) light through the leaf. As chlorophyll pigments absorb red light efficiently while allowing NIR light to pass through, the SPAD device generates an index value indicative of chlorophyll concentration (Mielcarek et al., 2018). When plants experience abiotic or biotic stress such as drought, salinity, nutrient deficiency, or pathogen attack, chlorophyll biosynthesis is often suppressed, leading to reduced SPAD values that can signal stress before any visible symptoms appear. This rapid, on-site diagnostic tool supports precision agriculture by enabling real-time tracking of photosynthetic efficiency and the early identification of physiological disruptions (Ojoatre, 2016). Its ease of use, portability, and capacity for high-throughput data collection make it essential for large-scale phenotyping, agronomic evaluations, and plant stress studies.

#### Electrical impedance tomography

Electrical Impedance Tomography (EIT) is an advanced and non-invasive imaging technique that farmers can utilize to assess the physiological stress of crops and plants. This method examines the internal distribution of electrical conductivity within plant tissues. The aforementioned process entails the precise arrangement of electrodes around plant structures, most commonly stems or leaves, through which low-amplitude alternating currents are systematically applied. A trained individual captures the resulting voltage measurements obtained from multiple electrode pairings at the periphery. The readings obtained are processed using inverse computational techniques, and the reason to do so is to reconstruct a high-resolution image capable of capturing spatial impedance variations (Corona-Lopez et al., 2019). Different research groups have focused on the fact that stress conditions, including drought, nutrient deficiencies, and pathogen attacks, alter physiological parameters such as water content, membrane stability, and ion movement, all of which are reflected in the impedance data. Thus, the EIT methodology is ideally suited for the continuous assessment of plant stress over extended periods. Additionally, some have noted that when this technology is integrated with WSNs, EIT enables scalable, on-site diagnostics across extensive agricultural systems, advancing precision agriculture by supporting the early detection and timely management of latent stress responses in crops (Basak and Wahid, 2022).

## Use of machine learning techniques for stress assessment and prediction

The implementation of Artificial Intelligence (AI), ML, and Deep Learning (DL) techniques in agricultural monitoring has greatly enhanced the accuracy and effectiveness of detecting both biotic and abiotic stressors in crops through real-time, non-destructive means. Conventional approaches rely largely on manual scouting and visual assessments, which are often constrained by limited spatial reach, interpretative bias, and delayed response times. In contrast, ML-based solutions utilize sophisticated computational algorithms and pattern recognition methods to analyze high-dimensional, heterogeneous data collected through advanced remote sensing platforms, such as RGB imaging, multispectral and hyperspectral sensors, thermal cameras, and LiDAR systems. **Table 3** outlines data types that are readily accessible and can be personally collected with minimal effort, through either destructive or non-destructive methods. These data types can be effectively utilized in ML algorithms to extract valuable insights for assessing stress in crops and plants, in addition to future stress prediction.

**Table 3:** Machine learning data types for crop and plant stress assessment.

S. no	Data type	Description	Suggested ML method
1	Chlorophyll Content	Indicates photosynthetic capacity, often measured via SPAD meters or sensors.	Random Forest or SVMs, for classification of stress levels based on chlorophyll variations.
2	Leaf Water Potential	Quantifies plant water status under varying field moisture conditions.	Gradient Boosting, which captures non-linear relationships in physiological water stress.
3	Malondialdehyde (MDA)	Lipid peroxidation marker indicating cell damage due to abiotic stress.	Decision Trees as an interpretable model of oxidative stress severity.
4	Proline Content	Osmoprotectant compound accumulation indicates drought or salinity stress.	Support Vector Machines, suitable for identifying osmotic stress patterns.
5	Air/Soil Temperature	Stress modeling based on field temperatures over time.	Linear Regression is suitable for modeling temperature stress relationships.
6	Growth Stage and Development	Helps contextualize when stress occurs during development.	Naive Bayes is a simple classifier based on phenological stage likelihoods
7	Irrigation/Fertilization Logs	Input data to relate management practices to stress outcomes.	Decision Trees relate input patterns to stress occurrence.
8	Yield Records	Reflects the cumulative impact of stress across the growing season.	Multiple Linear Regression predicts yield reduction
9	Genotypic Data	Genetic markers associated with stress tolerance traits.	XGBoost handles sparse, high-dimensional genomic data effectively.
10	Metabolomics Profile	Changes in metabolites such as sugars and amino acids reflect stress metabolism.	Random Forest is a dimensionality reduction method with robust classification.
11	Hyperspectral Images	High spectral resolution data, useful for detecting subtle physiological and biochemical changes.	CNNs or LSTMs handle high-dimensional spectral features.
12	LiDAR	Provides 3D canopy structure for stress-related morphological changes (height, density).	CNNs or RF model plant structural changes under stress.
13	Multispectral Images	Captures specific spectral bands for indices such as NDVI to assess a plant’s health.	SVMs classify stress levels based on spectral indices.
14	RGB Images	Standard RGB images, used to detect visible stress symptoms such as discoloration and wilting.	CNNs, effective in detecting visual stress symptoms.
**15**	Thermal Images	Measures canopy temperature variations indicating water stress and transpiration shifts.	CNNs or Regression Trees capture canopy temperature anomalies.

The preceding discussion shows that both destructive and non-destructive stress assessment methods can generate comprehensive datasets. These datasets can offer insights into plant health indicators and canopy-level physiological changes across multiple spectral bands, resolutions, and temporal scales. Once users collect these datasets, they can potentially detect stress conditions, including drought, nutrient deficiencies, pest attacks, and disease outbreaks. Supervised ML algorithms such as Support Vector Machines (SVMs), Random Forest (RF), Gradient Boosting Decision Trees (GBDT), and k-Nearest Neighbor (k-NN) are widely used by researchers around the world in several institutes for classification and regression tasks related to precision agriculture. These classification and regression methodologies can facilitate accurate mapping of the acquired data on different degrees of crop stress (Zubler and Yoon, 2020). Simultaneously, unsupervised ML techniques such as k-Means Clustering and Self Organizing Map (SOM) are employed to detect anomalies and reveal underlying structures within unlabeled datasets. To enhance predictive accuracy and computational efficiency, feature engineering methods including Principal Component Analysis (PCA), wavelet transforms, and texture-based feature extraction are used to condense high-dimensional data and emphasize key spectral or temporal traits. Additionally, ensemble modeling techniques and hybrid meta-learning frameworks are adopted to strengthen model robustness and ensure reliable performance across varying agro-ecological conditions (Karthickmanoj et al., 2021).

**Figure 8:**
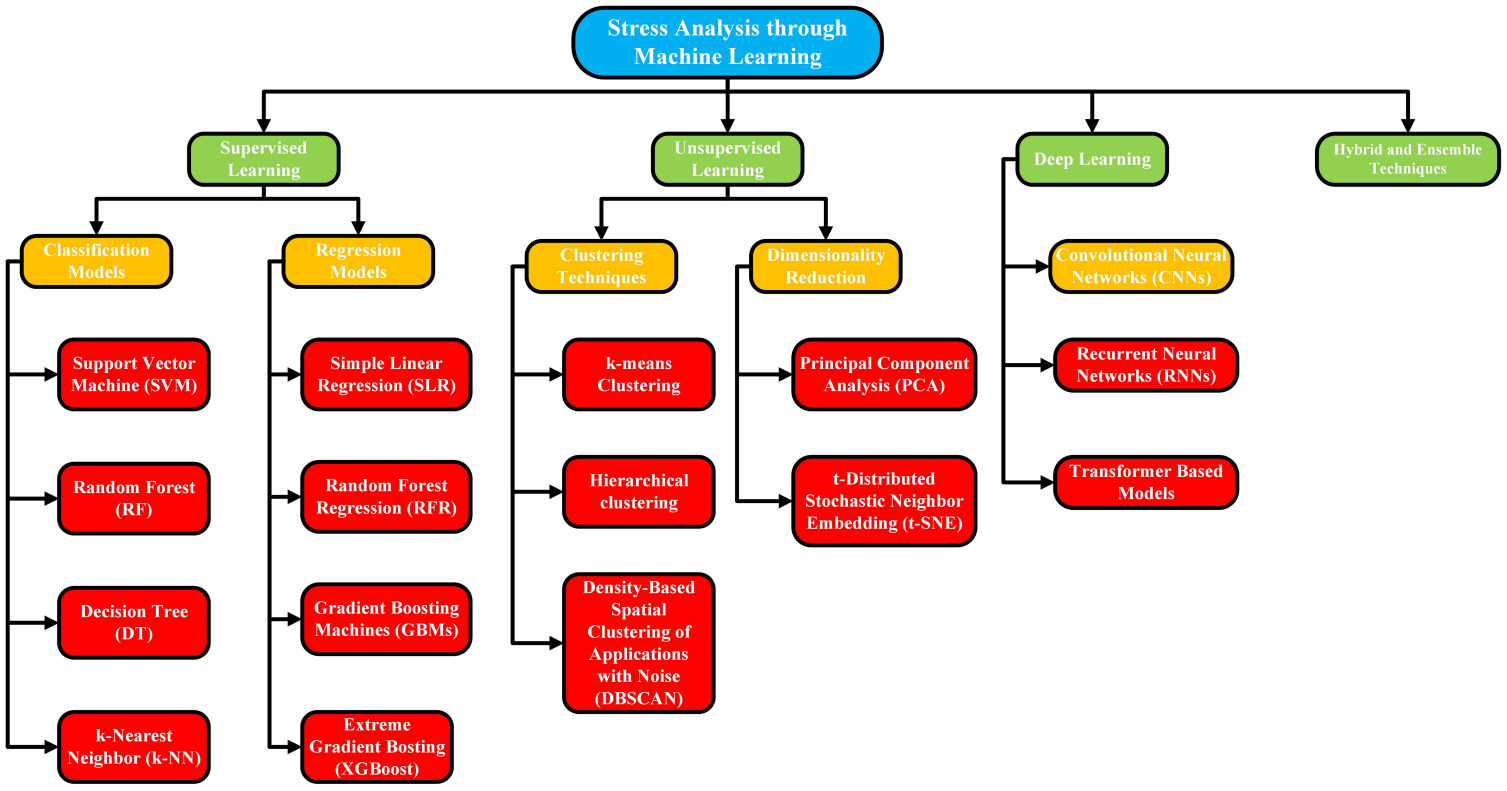
A flow chart of ML algorithms and their subcategories used for stress analysis in crops and plants.

DL techniques, with a focus on Convolutional Neural Networks (CNNs), provide a highly effective framework for the automated end-to-end extraction of features from image datasets, enabling layered analysis of spatial and spectral attributes linked to plant physiological conditions. Advanced CNN models such as the Residual Network (ResNet) and Inception have consistently delivered superior performance compared to other models in identifying crop stress, segmenting plant structures, and detecting disease lesions. Additionally, Recurrent Neural Networks (RNNs) and Long Short-Term Memory (LSTM) architectures are widely used for forecasting time series trends based on environmental sensor inputs and phenological data, supporting the creation of predictive models and early stress detection systems. Furthermore, attention-based mechanisms and transformer architectures are increasingly adopted to capture complex spatiotemporal relationships in large-scale agricultural datasets, thereby enhancing the accuracy and contextual relevance of model predictions. Additionally, the integration of AI and DL technologies with WSNs, UAVs, and cloud-based platforms enables the efficient and scalable acquisition, processing, and monitoring of real-time data (Gao et al., 2020). To further optimize performance and safeguard data privacy, edge computing and federated learning frameworks are being increasingly adopted, allowing for decentralized inference with reduced latency and enhanced data security. Reinforcement Learning (RL) is also being investigated for closed-loop optimization in precision agriculture, empowering autonomous systems to dynamically adjust their actions in response to evolving crop stress conditions. In **Figure 8** we presented a flow diagram designed to assist readers in easily identifying the most widely and commonly used ML algorithms for stress analysis in crops and plants, along with their respective subcategories.

### Supervised learning approaches

Supervised Learning (SL) methods, commonly employed by various researchers across the world can be broadly divided into two main categories (i) classification models and (ii) regression models. This section offers a concise overview of these categories, focusing on their roles in identifying, analyzing, and forecasting stress and related conditions in crops and plants.

#### Classification models

Over time supervised ML techniques, especially classification models, have become a core component of agricultural data analysis. Their effectiveness has been demonstrated by researchers and practitioners worldwide, consistently delivering valuable and beneficial outcomes. These models primarily offer critical solutions that can distinguish whether a crop or a plant is healthy or experiencing some form of stress that is hindering its growth. These models rely on labeled datasets, where class labels such as “stressed” or “healthy” are predefined, enabling the algorithm to learn discriminative patterns and establish predictive relationships between input features and output classes. The growing accessibility of high-resolution data from remote sensing platforms including multispectral, hyperspectral, and thermal imaging along with field physiological measurements, has significantly enhanced the accuracy, robustness, and scalability of these classification systems across diverse agricultural scenarios (Navrozidis et al., 2022).

- Support Vector Machines (SVMs) are a frequently utilized classification method that is highly effective for processing high-dimensional data and modeling intricate, non-linear patterns through kernel-based approaches. In plant phenotyping, SVMs have demonstrated robust performance in differentiating between biotic and abiotic stress by determining optimal hyperplanes that separate stressed and healthy plant instances based on inputs such as spectral reflectance, canopy temperature, and chlorophyll fluorescence. The underlying principle of margin maximization enhances the model’s generalization ability, making SVMs particularly suitable for the early detection of plant stress across diverse and dynamic environmental conditions (Islam et al., 2024).
- Random Forest (RF), a robust ensemble classification technique, constructs numerous decision trees during the training phase and determines the outcome by taking a majority vote from all tree outputs. It is particularly adept at modeling intricate, non-linear variable interactions, demonstrating high resilience to overfitting, and maintaining accuracy even when working with noisy or incomplete datasets. In the realm of agriculture, RF has been successfully utilized for detecting plant stress in diagnosing diseases using inputs such as leaf images and UAV-based spectral data, enabling reliable classification of issues such as nutrient deficiencies, leaf blight, and powdery mildew. Additionally, RF offers intrinsic measures of feature importance, helping to pinpoint the most influential spectral bands or physiological traits that drive accurate stress classification (Yang et al., 2021) (Gupta et al., 2023).
- Decision Trees (DTs), less complex than ensemble approaches, are valued for their clear structure and interpretability, which makes them ideal for use in agricultural decision support systems. They operate by systematically splitting the feature space using metrics such as entropy or the Gini index, resulting in a set of decision rules that classify stress conditions based on threshold values from sensor-derived features such as NDVI, PRI, or canopy temperature. While DTs are prone to overfitting, particularly with small datasets, their generalization performance can be significantly improved through techniques such as pruning and cross-validation (de Oliveira et al., 2023) (Yadav and Swarnkar, 2024).
- k-Nearest Neighbor (k-NN) determines the class of a new data point by identifying the k most similar instances within the training set and assigning the majority label among them, with similarity often calculated using distance measures, such as Euclidean Distance. While k-NN can be computationally demanding for large datasets, it remains highly effective for localized classification tasks. In agricultural contexts, it has been applied to visually classify stress conditions from leaf imagery and to delineate disease-affected zones in precision farming. Its nonparametric nature offers a key advantage, particularly in situations where the underlying data distribution is unknown or highly complex (Guo et al., 2021; Zahid et al., 2022).

Supervised classification methods such as SVMs, RF, DTs, and k-NN have collectively established a fundamental analytical foundation for the data-driven evaluation of plant and crop stress. Their integration with sensor technologies, UAV-based data acquisition, and cloud-enabled agricultural platforms supports the accurate, scalable, and real-time detection of stress in crops. This facilitates informed, proactive decision making, and efficient resource utilization, which promotes the progression of precision agriculture toward more sustainable and resilient food production systems. **Table 4** presents several real-world applications of ML models across a variety of crop types, aimed at achieving effective stress assessment.

**Table 4:** Real world implementations of ML models In crop and plant stress assessment.

S. no	Crop/Plant	Stress type	ML technique and data	Real-world implementation and impact	Reference
1	Greenhouse-grown tomatoes	Fertigation/heat stress	Gradient Boosting using leaf temperature, PRI, and climate sensors	The multi-sensor platform detects stress remotely in operational greenhouse settings	(Elvanidi and Katsoulas, 2022a)
2	Walnut orchard	Water stress	Random Forest regression based on UAV multispectral imagery	Achieved 85% classification accuracy at the plant level	(Wang and Jin, 2023)
3	Potato fields	Drought stress	Retina-UNet model based on aerial RGB+NIR images	Achieved a 0.74 dice score for distinguishing stressed vs. healthy potatoes	(Butte et al., 2021)
4	Agarwood (trees)	Pests and diseases	lightweight CNN based on leaf images	Achieved approximately 0.97 F1 score for various stress classes	(Shafik et al., 2025)
5	Cranberry bog	Sun exposure/heat stress	CNN for segmentation and an irradiance model from drone and sky imagery	Temperature prediction aids in irrigation scheduling	(Akiva et al., 2022)
6	Coffee leaves	Disease severity	CNN multi-task classification + severity estimation	Early detection and quantification of disease	(Esgario et al., 2020)

#### Regression models

Supervised ML regression models provide a technically advanced and scalable framework for quantifying stress severity in crops and plants, playing a pivotal role in precision agriculture. These models are trained on labeled datasets in which continuous output variables representing measurable indicators of stress severity, such as the extent of chlorosis, leaf water potential, and deviations in canopy temperature, are linked with a wide array of input features. These features are extracted from diverse data sources, including multispectral and hyperspectral imagery captured by UAVs, thermal imaging systems, soil moisture probes, and weather-related parameters (Asaari et al., 2022). The primary objective of supervised regression is to construct a functional mapping that can effectively capture and predict the severity of biotic or abiotic stress impacting plant health by representing a high-dimensional vector comprising environmental and physiological variables. Among the various regression algorithms used for this application, Simple Linear Regression (SLR), Random Forest Regression (RFR), Gradient Boosting Machines (GBMs), and Extreme Gradient Boosting (XGBoost) are particularly noteworthy, each offering unique modeling structures, learning strategies, and levels of predictive performance.

- Simple Linear Regression (SLR) is the most fundamental parametric regression technique, based on the premise of a linear association between the dependent variable, representing stress severity, and a group of independent input features. It determines the model coefficients *β_i_* using the linear formulation *y* = *β*_0_ + Σ*β_i_x*_i_ + *ϵ_r_*, where *ϵ* epsilon represents the stochastic error component that accounts for residual variability. Although this method offers strong interpretability and low computational complexity, its predictive performance is often limited in complex agricultural environments, as it fails to model the nonlinear relationships and higher-order interactions inherent in plant physiological dynamics (Behmann et al., 2015).
- Random Forest Regression (RFR) is a nonparametric ensemble technique that constructs numerous independent decision trees through bootstrap aggregating to enhance predictive performance and minimize variance. Each tree is trained on a bootstrapped sample of the data that utilizes a randomly selected subset of features at each decision split. The overall prediction is computed by averaging the outputs of all the trees in the ensemble. This framework allows the model to effectively learn complex nonlinear dependencies among variables such as vegetation indices (e.g., NDVI, PRI), thermal stress metrics (e.g., Crop Water Stress Index (CWSI)), and topographic attributes, while inherently controlling for overfitting. Moreover, RFR provides feature importance scores that are valuable for identifying key stress-related factors and for ranking input variables by relevance (Yang et al., 2021). Nonetheless, the method may encounter scalability challenges when applied to high-dimensional remote sensing data, as the computational and memory requirements for training and storing a large number of deep trees can be substantial (Hasanuzzaman et al., 2020).
- Gradient Boosting Machines (GBMs) are iterative ensemble models in which each base learner, usually a shallow decision tree, is trained in sequence to reduce the negative gradient of a selected loss function, most often the mean squared error. This additive learning strategy progressively refines the model by correcting the residual errors from previous iterations. GBMs excel at modeling the complex, nonlinear associations between the temporal or spectral input features of the plant stress responses. Nevertheless, they can be prone to overfitting, particularly when regularization is inadequate or when deep trees are used. Achieving robust generalization typically requires careful tuning of key hyperparameters, such as the learning rate, maximum tree depth, and number of boosting iterations (Elvanidi and Katsoulas, 2022b).
- Extreme Gradient Boosting (XGBoost) is a high-performance, optimized evolution of GBMs, tailored to deliver enhanced scalability, increased computational speed, and stronger regularization. It employs a second-order Taylor Series expansion to improve gradient optimization accuracy and incorporates penalties to effectively manage model complexity and reduce the likelihood of overfitting. XGBoost also supports efficient handling of sparse data and enables parallel tree construction, making it particularly well-suited for large, high-dimensional agricultural datasets (Huber et al., 2022). Its high predictive accuracy, combined with its compatibility with advanced interpretability techniques such as Shapley Additive Explanations (SHAP) values and gain-based feature importance, makes it a preferred algorithm for modeling and predicting plant stress severity across varying environmental conditions.

These supervised regression models collectively provide a solid foundation for predictive analytics in agricultural stress monitoring. When combined with UAV-based imaging systems, ground-level sensor networks, and cloud-edge computing infrastructure, they enable precise spatial and temporal predictions of crop stress severity. This capability supports key precision agriculture strategies, including site-specific irrigation, efficient pesticide application, and the early detection of plant health issues. The choice of an appropriate algorithm is typically influenced by the balance between model transparency, computational efficiency, and the complexity of feature-target relationships. Within modern agronomic decision support systems, these regression approaches serve as the core of scalable, data-driven solutions aimed at promoting sustainable crop production and optimizing yields under varying environmental and biological stress conditions. **Table 5** presents a comparative analysis of the previously discussed supervised ML algorithms, along with their reported performance metrics as documented in various research studies.

**Table 5:** Comparative analysis of supervised ml algorithms used for stress analysis and prediction in crops and plants.

S. no	Metric	SVMs	RF	DTs	k-NN	SLR	RFR	GBMs	XGBoost
1	Accuracy (%)	88–93	90–95	82–88	85–91	76–82	90–94	92–96	93–97
2	Precision (%)	87–92	89–94	80–86	83–89	74–80	89–93	91–95	92–96
3	Recall (%)	85–91	90–96	78–84	82–90	72–78	90–95	92–97	93–98
4	F1-Score (%)	86–92	89–95	79–85	83–89	73–79	89–94	91–96	93–97
5	Training Time	Medium	Fast	Very Fast	Slow	Very Fast	Fast	Medium	Medium
6	Inference Time	Fast	Fast	Very Fast	Medium	Fast	Fast	Medium	Medium
7	Model Complexity	High	Medium	Low	Low	Very Low	Medium	High	High
8	Noise Robustness	Medium	High	Low	Medium	Low	High	High	Very High
9	Scalability	Medium	High	Medium	Low	Low	High	High	Very High
10	Interpretability	Low	Medium	High	Low	High	Medium	Low	Medium

### Unsupervised learning approaches

Unsupervised learning approaches have also demonstrated significant utility in precision agriculture by identifying natural clusters of stressed and healthy plants without requiring labeled datasets. In contrast to supervised learning, which necessitates predefined labels for training, unsupervised approaches extract hidden structures from unlabeled data, making them particularly valuable in agricultural scenarios where manual labeling is labor-intensive and difficult to scale (Zubler and Yoon, 2020; Lu et al., 2023). These clustering techniques categorize plant data based on inherent similarities across features such as spectral reflectance, thermal output, chlorophyll levels, morphological characteristics, and physiological indicators, typically gathered through UAV imagery, ground-based sensors, or remote sensing technologies.

#### Clustering techniques

Clustering techniques play a vital role in agriculture by detecting crop and plant stress through the identification of natural groupings within unlabeled data. These unsupervised learning methods evaluate patterns of features such as spectral reflectance, thermal data, and vegetation indices to differentiate stressed plants from healthy ones. Algorithms such as k-means, hierarchical clustering, and Density Based Spatial Clustering of Applications with Noise (DBSCAN) effectively group plant populations based on physiological characteristics, enabling the timely detection of stress factors such as nutrient shortages, water stress, and disease. Offering spatially detailed insights without requiring labeled inputs, clustering aids in implementing precise interventions and strengthens decision-making processes to enhance crop health and overall agricultural productivity.

- K-Means Clustering is widely used due to its ability to divide a dataset into *k* distinct clusters by minimizing variation within each group. It operates through an iterative process that assigns data points to the nearest centroid and continuously refines the cluster centers, effectively highlighting well-defined zones that may correspond to healthy, moderately stressed, or severely stressed vegetation. Its straightforward implementation and ability to scale efficiently make it well-suited for processing high-resolution agricultural datasets. However, a notable drawback is the need to specify the number of clusters *k* in advance, which can be challenging in dynamic and variable stressed field conditions (Dickinson et al., 2018; Javidan et al., 2023).
- Hierarchical Clustering is a well-established method that does not necessitate predefining the number of clusters. It builds a hierarchical tree structure by either gradually merging individual data points or successively splitting a single overarching cluster, based on calculated distance metrics. This technique offers a flexible and intuitive framework for capturing the progression of plant stress across a field, particularly in scenarios where stress levels fluctuate over time or vary across spatial regions (Pantazi et al., 2017) (Frank et al., 2021).
- Density Based Spatial Clustering of Applications with Noise (DBSCAN) is a powerful solution for examining intricate, non-linearly separable stress distributions in noisy agricultural datasets. It forms clusters by locating densely packed data points, while designating low-density areas, which typically correspond to highly stressed or anomalous crop regions as outliers. Its ability to detect clusters of irregular shapes and filter out noise makes it particularly effective in diverse agricultural environments, where stress patterns deviate from standard spatial structures (Liu et al., 2020; Zhao et al., 2024).

#### Dimensionality reduction techniques

The performance of unsupervised clustering algorithms can be significantly enhanced through the use of dimensionality reduction techniques such as PCA and t-Distributed Stochastic Neighbor Embedding (t-SNE) (Zhu et al., 2021; Dutağaci, 2023). These approaches simplify complex, high-dimensional sensor data such as multispectral or hyperspectral imagery, leading to improved clustering precision and more intuitive visual interpretation. In agricultural practice, the clustered outputs can be mapped geospatially to identify spatial stress patterns within fields, enabling precision interventions such as site-specific irrigation, targeted pesticide application, or soil treatment. Additionally, by integrating these clustering methods with edge computing platforms, real-time analysis can be performed directly on UAVs or sensor nodes, delivering immediate, field-level insights and decision support. As agricultural data continues to expand in both scale and complexity, unsupervised clustering provides a flexible, scalable, and label-free solution for extracting meaningful insights from raw sensor inputs, thereby supporting sustainable and data-informed farming strategies tailored to the dynamic variability of crop conditions. **Table 6** presents a comparative analysis of the previously discussed unsupervised ML algorithms, along with their reported performance metrics as documented in various research studies.

**Table 6:** List Of Unsupervised ML techniques used commonly to analyze and predict stress in crops and plants.

S. no	Metric	k-means clustering	Hierarchical clustering	DBSCAN	PCA	t-SNE
1	Accuracy (%)	70–80	75–85	70–90	78–88	80–90
2	Precision (%)	68–78	72–82	75–88	76–86	78–89
3	Recall (%)	65–75	70–80	78–90	77–87	80–92
4	F1-Score (%)	66–76	71–81	76–89	76–87	79–90
5	Training Time	Fast	Slow	Medium	Fast	Slow
6	Inference Time	Fast	Fast	Very Fast Medium	Medium	Medium
7	Model Complexity	Low	Low	Low	High	Very High
8	Noise Robustness	Low	Medium	High	Medium	Medium
9	Scalability	High	Low	Medium	High	Low
10	Interpretability	Moderate	High	Moderate	High	Low

### Deep learning methods

DL methods are swiftly emerging as a revolutionary approach in the field of agriculture, particularly for detecting and analyzing stress in crops and plants. By leveraging vast datasets generated from advanced imaging systems and environmental sensors, these models can effectively recognize, categorize, and anticipate various stress conditions resulting from environmental, biological, or chemical factors. They enable automated evaluation of plant health by examining key visual features such as leaf color, structure, and surface texture, allowing for the early detection of issues such as drought, pest attacks, and nutrient deficiencies. The following subsection presents a concise overview of the most commonly employed DL techniques for analyzing stress in crops and plants.

- Convolutional Neural Networks (CNNs) are widely utilized in spatial assessments of plant stress due to their strong capability to learn layered feature representations from visual data. When applied to RGB, multispectral, or hyperspectral imagery, CNNs can autonomously extract spatial features associated with plant structure, color, and texture. These are key indicators for detecting stress symptoms such as chlorosis, wilting, and necrotic damage. To enhance the accuracy of tasks such as semantic segmentation, object detection, and stress classification, advanced CNN frameworks such as U-Net, ResNet, and Efficient Neural Network (ENet) are frequently employed in agricultural imaging. For instance, a CNN trained on NDVI-derived imagery can accurately map drought-impacted areas with fine spatial resolution (Chandel et al., 2021). Additionally, CNN architectures are often augmented with components such as attention mechanisms or spatial pyramid pooling layers to improve their contextual understanding and adaptability to varying spatial scales (An et al., 2019).
- Recurrent Neural Networks (RNNs), particularly LSTM models, excel at capturing temporal variations within sequential agricultural datasets regarding the stress on crops and plants. These models are well equipped to manage complex, time-dependent, and non-linear data, making them particularly suitable for evaluating long-term datasets such as phenological timelines, time series vegetation indices (e.g., NDVI, Extended Visual Information (EVI)), and environmental metrics such as humidity and temperature (Ali et al., 2024). LSTMs leverage internal memory units to retain important temporal information across extended timeframes, which is critical for anticipating stress conditions before they escalate. For example, LSTM networks can predict upcoming water stress by analyzing historical patterns in soil moisture and climatic conditions. Furthermore, integrated CNNs based on LSTM architectures are gaining popularity for spatiotemporal analysis. In this context, CNNs extract spatial features from image data, while LSTMs model the evolution of stress indicators across time (Bounoua et al., 2024).

Originally introduced for natural language processing, transformer-based models have recently been adopted for agricultural stress analysis due to their effectiveness in capturing long-range dependencies and contextual cues via self-attention mechanisms. Vision Transformer (ViT) and hybrid CNN transformer architectures demonstrate strong performance in identifying broad spatial patterns within plant imagery, making them particularly valuable for detecting widespread or subtle stress manifestations across large agricultural areas (Thokala and Doraikannan, 2023). Unlike CNNs, which focus on localized receptive fields, transformers evaluate all image segments simultaneously, enabling the detailed modeling of spatially complex and heterogeneous stress conditions. Furthermore, transformers excel at fusing multimodal data sources including visual, spectral, thermal, and climatic inputs. This is especially advantageous for diagnosing compound stress events, such as the co-occurrence of drought and nutrient deficiency, where understanding intricate feature interdependencies is critical for an accurate assessment (Koike et al., 2024). **Table 7** presents a comparative analysis of the previously discussed DL algorithms, along with their reported performance metrics as documented in various research studies.

**Table 7:** List of deep learning techniques commonly used to analyze and predict stress in crops and plants.

S. no	Metric	CNNs	RNNs	LSTM	Transformer-based models
1	Accuracy (%)	88–94	85–91	87–93	92–98
2	Precision (%)	87–93	84–89	86–92	91–97
3	Recall (%)	86–92	83–88	85–91	90–96
4	F1-Score (%)	86.5–93	83.5–89	85.5–92.5	91–96.5
5	Training Time	Medium	Medium	High	Very High
6	Inference Time	Fast	Medium	Medium	Fast (if optimized)
7	Model Complexity	Moderate	Moderate	High	Very High
8	Noise Robustness	Moderate	Moderate	High	Very High
9	Scalability	High	Moderate	Moderate	High
10	Interpretability	Excellent (image-based)	Moderate	Moderate	High with attention mechanisms

### Hybrid and ensemble learning approaches

Hybrid and ensemble learning methods have proven to be highly effective in advancing stress detection in crops and plants. These methods bolster model resilience, enhancing prediction accuracy and improving generalization, particularly in agricultural settings where data may be noisy, incomplete, or influenced by fluctuating environmental conditions. The literature indicates that these techniques can improve the performance of various models used by different researchers for different agriculture-dependent applications. These methods include CNNs, which can eliminate important, less frequent spatial features from image-based data. Once these features are extracted, then they can be merged with conventional classifiers such as RF to process data with a high level of dimensionality. We have found that in the integrated framework, CNNs can independently learn layered representations of stress-related features from RGB or multispectral images. Once learned, this information is utilized by RF classifiers for precise and interpretable decision-making. This synergistic model architecture enables multiscale feature representation, leading to improved classification outcomes in complex field conditions involving symptoms such as chlorosis, disease spots, and drought-related morphological alterations (Azrai et al., 2024). Moving forward, we examined ensemble learning strategies and found that they can improve the analytical framework. This is achieved by averaging the predictions of many different base models, which ultimately helps minimize variance, correct bias and enhance the ability of the model to address noisy or inconsistent data. This inconsistency in the data can be attributed to human error, negligence, or malfunctioning data-collecting sensors. Bagging techniques such as those employed in RF algorithms leverage bootstrap resampling to generate varied training subsets and aggregate their predictions, which increases model reliability and decreases variability. On the other hand, boosting methods such as Adaptive Boosting (AdaBoost), GBMs, and XGBoost build models sequentially, adjusting the focus toward misclassified samples in each iteration. This adaptive learning process improves the model’s sensitivity to subtle or complex stress indicators in crops and plants. Stacking, a sophisticated ensemble approach that integrates diverse base learners such as SVMs, DTs, and Neural Networks (NNs) by training a meta-model that learns the optimal combination of their outputs, resulting in superior generalization across diverse and intricate datasets. These ensemble frameworks are especially useful in the context of agricultural stress assessment because data variability and non-linear relationships are common. Overall, hybrid and ensemble learning methods provide scalable, flexible, and highly accurate computational solutions for precise stress identification and classification in precision agriculture, supporting informed decision making and efficient resource management (Batool et al., 2022).

**Table 8** presents a comparative overview that consolidates key insights regarding the advantages and disadvantages of destructive, non-destructive, and ML-based methods. This table is designed to offer readers a concise and accessible summary.

**Table 8:** Comparison of destructive, non-destructive, and ML-based methods in crop and plant stress analysis.

S. no	Method type	Advantages	Limitations
1	Destructive Methods	• Direct measurement of internal biochemical and physiological parameters. • High accuracy in controlled conditions. • Useful for calibration and validation of remote methods.	• Invasive: results in the damage or death of sampled plants. • Labor-intensive and time-consuming. • Limited scalability for large fields.
2	Non-Destructive Methods	• Preserves plant integrity for continuous monitoring. • Enables high-frequency data collection. • Integrates with sensors and UAVs for real-time assessment.	• May have limited depth of internal physiological insight. • Requires calibration against destructive methods. • Equipment can be expensive.
3	ML-Based Methods	• Enables large-scale, automated stress detection. • Learns complex patterns from diverse data sets. • Suitable for predictive modeling.	• Requires large, labeled datasets for training. • May lack interpretability. • Performance is sensitive to data quality and domain variations.

### Comparative results

**Figure 9:**
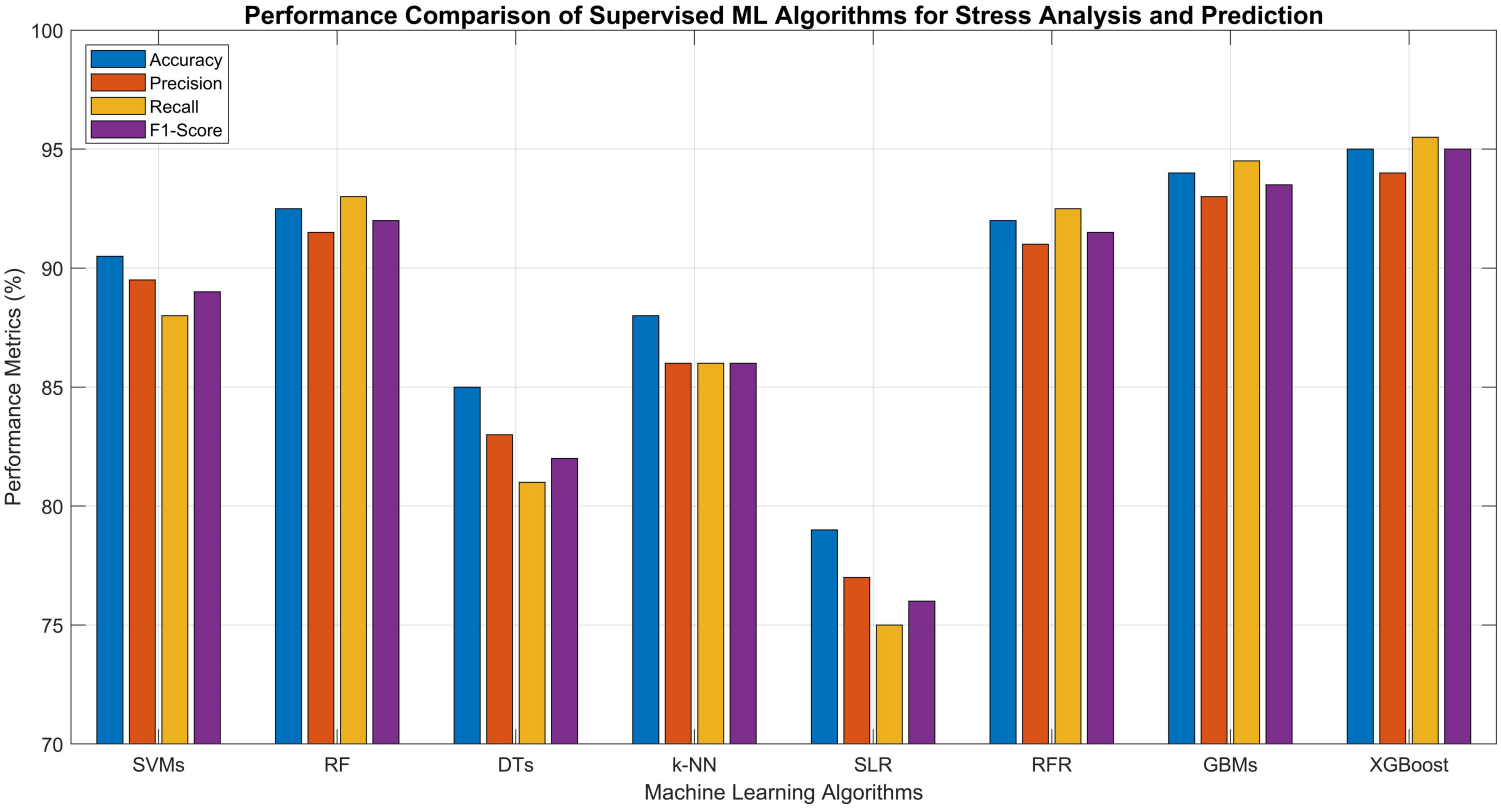
Comparative analysis of commonly employed supervised ml techniques for stress assessment in crops and plants.

As shown in **Figure 9** the results provide a detailed assessment of various supervised ML algorithms chosen by different researchers from different institutions around the world for the evaluation of stress-based signs in crops and plants. The list of methodologies includes SVMs, RF, DTs, k-NN, SLR, RFR, GBMs, and XGBoost. While evaluating data regarding various stress types, users incorporated key performance indicators such as accuracy, precision, recall, and F1-score, which ultimately helped them enable a comprehensive, multi-metric comparison of each model’s classification effectiveness within complex agroecological environments.

XGBoost and GBMs demonstrated exceptional predictive capabilities, consistently achieving mean accuracies and F1 scores exceeding 92%. Their strong performance stems from advanced gradient boosting mechanisms which iteratively minimize prediction errors by refining residuals throughout the training process. Moreover, these models excel at capturing complex non-linear relationships and hierarchical feature structures, making them particularly well-suited for analyzing high-dimensional sensor data and hyperspectral images. RF and RFR also delivered strong performance and excellent generalization by employing ensemble learning and bootstrap aggregation techniques. These methods effectively address prevalent issues in agricultural stressor datasets, such as overfitting, noise, and class imbalance, thereby enhancing model reliability and stability. SVMs performed well in high-dimensional feature spaces. However, its effectiveness depends heavily on appropriate kernel selection and meticulous parameter tuning. In contrast, k-NN faced challenges with scalability and is highly sensitive to irrelevant or noisy features. DTs were found to be advantageous for their fast training and ease of interpretation; however, they often struggled with high variance, leading to suboptimal performance. SLRs were limited by their assumption of linearity, which is inadequate for modeling the complex non-linear interactions typically observed in stress-related data for crops and plants.

**Figure 10:**
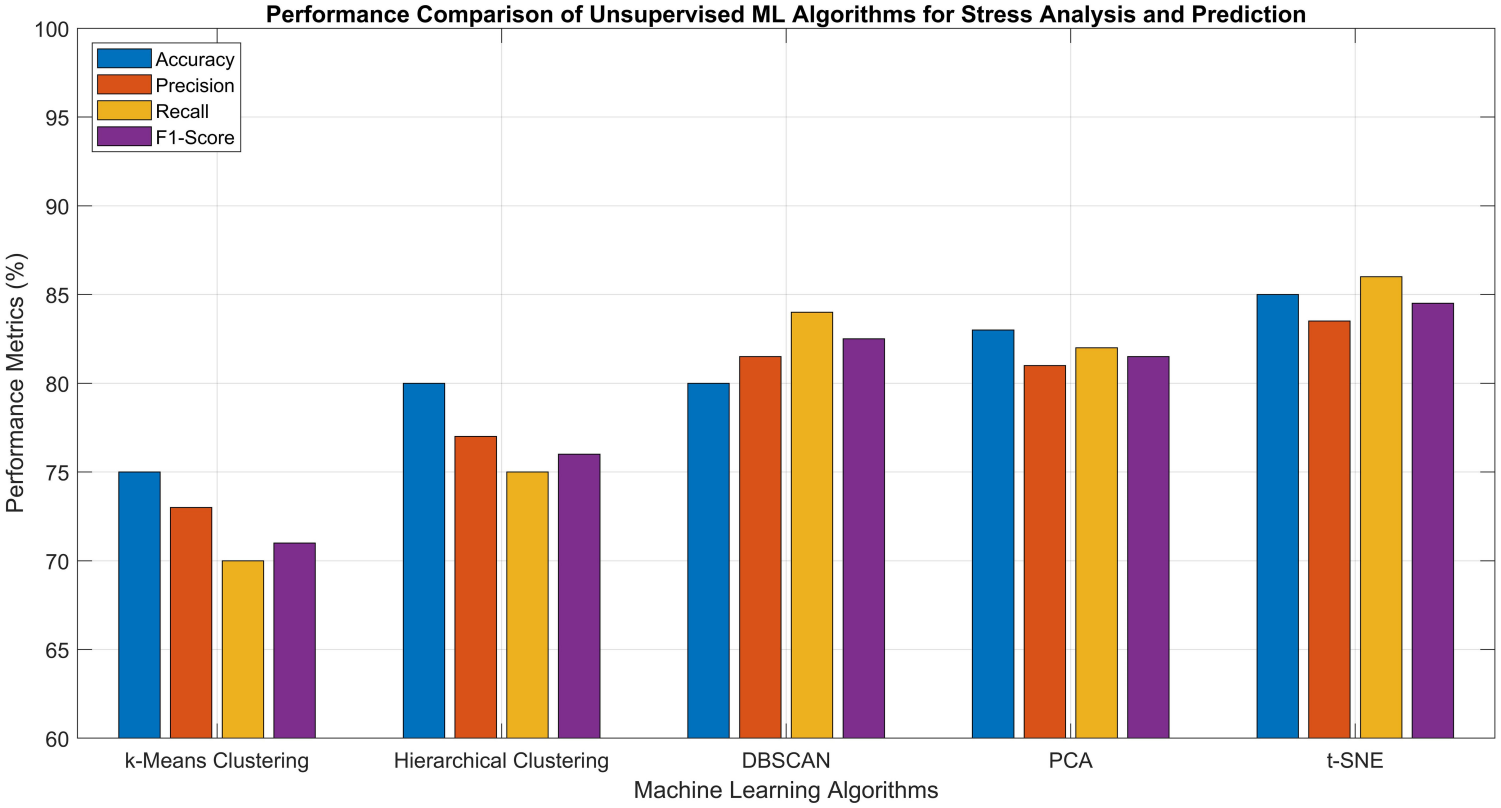
Comparative analysis of commonly employed unsupervised ML techniques for stress assessment in crops and plants.

**Figure 10** shows a bar graph that offers a comparative analysis of commonly applied unsupervised ML methods. These include k-Means Clustering, Hierarchical Clustering, DBSCAN, PCA, and t-SNE. These analyses are based on four core performance metrics: Accuracy, Precision, Recall, and F1-Score. These performance values represent averaged estimates obtained from detecting and classifying stress in crop- and plant-based datasets.

The graph indicates that t-SNE and PCA, when paired with clustering algorithms, delivered the strongest overall performance across the four key metrics. Notably, t-SNE showed impressive results, with an accuracy of nearly 85%, a recall of approximately 86%, and an F1-score close to 84.5%, demonstrating its remarkable capability to preserve local relationships and uncover subtle stress patterns in complex, high-dimensional crop and plant datasets, such as hyperspectral imagery. Similarly, PCA yielded solid performance, achieving an average accuracy of approximately 83% along with stable precision and recall values, emphasizing its utility in reducing dimensionality and effectively organizing feature space prior to clustering. DBSCAN showed competitive performance, with a particularly strong recall of 84% and an F1-score of 82.5%, highlighting its capability to effectively detect irregularly shaped stress patterns and manage noise, both of which are common in real-world agricultural data. In contrast, Hierarchical Clustering delivered steady, moderate performance across all evaluation metrics, making it well-suited for datasets with clear structural organization and gradual stress variations. On the other hand, k-Means, while highly efficient and scalable, registered relatively lower scores especially in terms of recall and F1-score. This is primarily due to its reliance on initial centroid selection and its limitation to spherical cluster assumptions.

**Figure 11:**
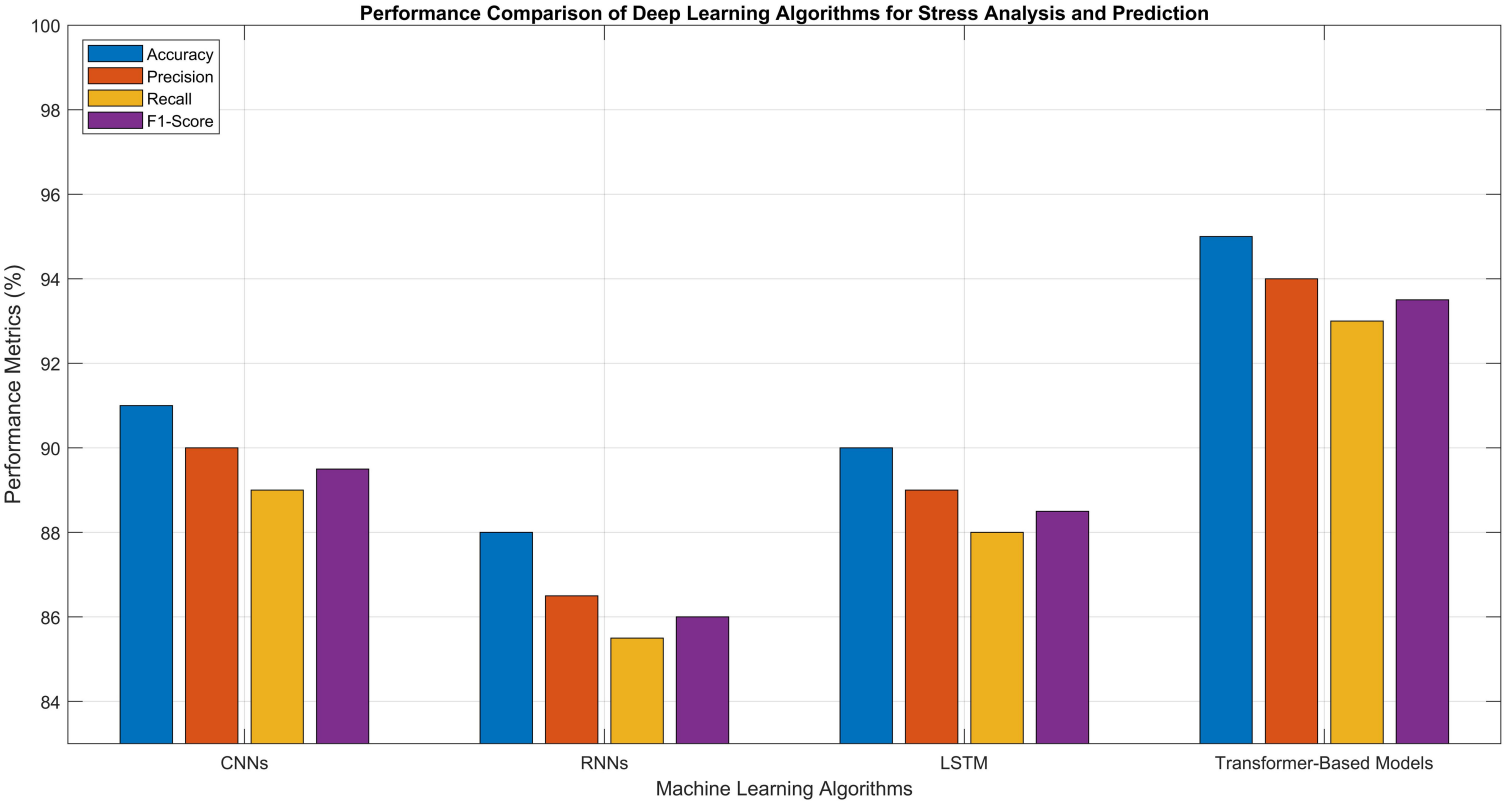
Comparative analysis of commonly employed deep learning algorithms for stress assessment in crops and plants.

The bar graph in **Figure 11** illustrates a comparative analysis of the selected DL algorithms that are CNNs, RNNs, LSTM networks, and Transformer-based models, within the scope of crop and plant stress identification and early stress forecasting. The assessment is based on four key performance indicators: Accuracy, Precision, Recall, and F1-Score, which together provide a comprehensive measure of each model’s classification, effectiveness and generalization ability.

Transformer-based models produced the strongest overall results, with average performance metrics falling between 93% and 95%. Utilizing self-attention mechanisms and parallel processing capabilities, these models showed that they were highly effective at capturing long-range interactions and contextual dependencies within diverse, multimodal agricultural stress-based datasets. Their elevated accuracy and recall values reflect a strong ability to detect stress indicators, particularly in cases involving diverse data sources, such as hyperspectral imaging, sequential sensor data, and environmental information. CNNs consistently performed well across all evaluation metrics, demonstrating strong capability to extract spatial features from image-based datasets. Their convolutional layer architecture excels at identifying localized patterns, making them particularly effective for detecting visual stress symptoms such as chlorosis, necrosis, and pest-related damage. LSTM networks outperformed standard RNNs by preserving long-term dependencies, resulting in more accurate and consistent predictions for sequential and time-dependent data. Their architecture incorporates gated memory units that effectively address the vanishing gradient issue, making them especially suitable for modeling the temporal progression of plant stress conditions. Conversely, the conventional RNNs showed relatively weaker performance, particularly in terms of recall and F1-score, due to their limited memory capabilities and vulnerability to gradient degradation, which limited their ability to learn from longer input sequences.

However a major technical challenge in implementing ML in agriculture is the limited availability of high-resolution, accurately annotated datasets, which are critical for developing robust and high-performance models. Supervised learning techniques especially advanced DL models such as CNNs, ViT, and GNNs require extensive, diverse datasets to ensure reliable generalization across different crop varieties and varied agro-ecological environments. However, acquiring such data is often obstructed by temporal and spatial inconsistencies, sensor calibration discrepancies, and the lack of standardized annotation frameworks. These obstacles result in insufficient data quality and quantity, limiting the scalability and adaptability of ML models in real-world field conditions. Moreover, the computational intensity of state-of-the-art ML architectures poses a further constraint, particularly when targeting deployment in edge computing systems with limited resources. DL models often demand significant computational infrastructure, including Graphics Processing Unit (GPU) acceleration and high memory capacity, during both the training and inference phases. These hardware requirements make real-time implementation in agricultural settings difficult, especially in environments with limited energy and processing capabilities. To address this, it is essential to investigate model optimization strategies such as lightweight network design, pruning, quantization, and knowledge distillation. These strategies all aim to improve computational efficiency without significantly affecting model accuracy. Scalability also presents algorithmic challenges, particularly across diverse agro-ecosystems, which are characterized by variations in crop physiology, stress conditions, and field management practices. These factors contribute to domain shift, reducing the effectiveness of models trained under controlled conditions when applied in the field. To overcome this, advanced techniques such as domain adaptation, transfer learning, and federated learning offer promising solutions to improve model robustness and adaptability in heterogeneous agricultural environments.

## Benefits of stress to plants and crops: a brief discussion

A plant that has never encountered stress would be as ill-equipped as a person who has never faced adversity. The growth and development of all living organisms, including plants and animals, are profoundly shaped by continual exposure to stress throughout evolutionary processes, ontogenetic stages, and individual life cycles. However, research on plant stress has largely focused on alleviating its harmful consequences, with minimal attention given to the scientifically sound approaches of acknowledging and leveraging the positive effects of stress on plants and crops.

In agricultural science, plant stress is typically linked to reduced productivity and physiological strain. However, when plants are subjected to moderate or controlled stress stemming from abiotic sources such as drought, salinity, temperature fluctuations, and nutrient limitations, or biotic challenges such as pests and pathogens, this can provoke adaptive morphological, physiological, and molecular changes that bolster plant vigor and resilience. In fact, a well-established response to such stresses is the enhanced synthesis of secondary metabolites (Godoy et al., 2021). These compounds, such as phenolics, flavonoids, alkaloids, and terpenoids, play essential roles in defending against environmental threats and can also mitigate oxidative damage. From an agronomic standpoint, it is obvious that the accumulation of these metabolites significantly improves crop quality traits, including flavor profile, antioxidant potential, and therapeutic value. For example, research done on viticulture has demonstrated that water stress is responsible for elevating anthocyanin levels in grape skins, which helps in contributing to superior sensory characteristics and chemical composition in wine (Arias et al., 2022).

In addition, plants exposed to early or repeated stress events often develop a form of stress resilience, known as stress priming or stress memory. This adaptive response is characterized by epigenetic alterations and gene expression reprogramming, both of which enable faster and more effective reactions to subsequent stress challenges. Key physiological adjustments include enhanced root development, refined stomatal regulation, and improved osmotic control: these support better water use efficiency and greater drought tolerance. Complementary morphological changes such as a reduced leaf area index, increased accumulation of cuticular wax, and modified leaf orientation also play a crucial role in reducing transpirational water loss and mitigating photoinhibition under conditions of intense sunlight or elevated temperatures (Gowtham et al., 2022). In the rhizosphere, abiotic stress can reshape the profile of root exudates, encouraging beneficial associations between plants and soil microorganisms. For example, under nutrient-poor conditions, roots release signaling compounds that attract arbuscular mycorrhizal fungi and nitrogen-fixing bacteria, thereby enhancing nutrient uptake and reducing dependence on chemical fertilizers. In saline soils, stress-tolerant endophytes and rhizobacteria help maintain ion balance and regulate antioxidant enzyme activity, thereby boosting the plant’s salt tolerance. These symbiotic relationships highlight the essential role of plant microbiome interactions in facilitating stress resilience and promoting sustainable agricultural practices (Solomon et al., 2023).

Additionally, deliberately managed stress applications have been used to fine-tune source-to-sink interactions, directing assimilates toward enhancing reproductive development. In fruit crops, for instance, Regulated Deficit Irrigation (RDI) has demonstrated success in elevating sugar content, increasing dry matter levels, and extending postharvest longevity without negatively affecting overall yield. These stress-induced shifts in metabolic allocation hold significant potential for enhancing the commercial quality and storage durability of perishable produce (Yang et al., 2022) (Chen et al., 2023). Finally, the transgenerational impacts of stress arising from genetic, epigenetic, and physiological changes can lead to improved seed vigor, consistent germination, and greater stress tolerance in the next generation. These inherited adaptive traits are being increasingly utilized in breeding initiatives aimed at developing climate-resilient crop varieties (Louis et al., 2023; Nguyen et al., 2022).

## Conclusion

In this comprehensive review we have tried to emphasize the complexity and significance of detecting abiotic and biotic stressors in crops, along with advancements in technologies specifically designed for their assessment. As time passes, the agricultural sector faces mounting pressures from climate change, environmental degradation, and human-driven influences. Therefore, the demand for swift and accurate stress detection has become critical. When crops and plants experience stress, they exhibit various stress indicators that can be categorized as visual, physiological, biochemical, or molecular in nature. Although conventional destructive methods have yielded reliable information over time, they are gradually being replaced by cutting-edge non-destructive techniques and remote sensing tools that offer continuous, large-scale monitoring without harming plant structures. The integration of ML into these platforms is a major breakthrough, as it enables automated, high-throughput detection, classification, and predictive modeling of stress responses. These advancements facilitate better decision-making, optimize resource use, and contribute to the evolution of precision agriculture, while also supporting the development of stress-resilient crop varieties. Looking ahead, sustained interdisciplinary collaboration and technological innovation will be pivotal in addressing future agricultural challenges and securing the global food supply. Our effort through this review offers critical insights for scientists, agronomists, and policymakers seeking to boost crop yields through advanced stress assessment and responsive management practices.

Future research efforts should focus on the synergistic integration of diverse remote sensing inputs including hyperspectral, thermal, LiDAR, and chlorophyll fluorescence data acquired from UAVs and satellite-based platforms with advanced deep learning frameworks such as Vision Transformers and Graph Neural Networks to enable precise, high-resolution spatiotemporal mapping of crop stress dynamics. Equally important is the advancement of interpretable AI through the adoption of explainable ML approaches, e.g., SHAP and Local Interpretable Model agnostic Explanations (LIME), which are essential for transparent, data-driven decision making. In parallel, incorporating multi-omics datasets spanning genomic, transcriptomic, and proteomic layers into phenomics-centric pipelines can accelerate the identification and validation of stress-resilient genotypes. To ensure methodological consistency and broad applicability, the establishment of unified stress quantification standards and interoperable data ontologies is vital for enhancing reproducibility, scalability, and cross-environmental robustness in precision phenotyping frameworks.

## Glossary

AAS: Atomic Absorption Spectrophotometry
ABA: Abscisic Acid
AdaBoost: Adaptive Boosting
AI: Artificial Intelligence
APX: Ascorbate Peroxidase
ATP: Adenosine Triphosphate Production
CAT: Catalase
CNNs: Convolutional Neural Networks
CTF: Controlled Traffic Farming
CWSI: Crop Water Stress Index
CWSI: Crop Water Stress Index
DAB: Diaminobenzidine
DBSCAN: Density-Based Spatial Clustering Of Applications With Noise
DL: Deep Learning
DMSO: Dimethyl Sulfoxide
DNA: Deoxyribonucleic Acid
DNase I: Deoxyribonuclease I
DRE/CRT: Dehydration-Responsive Element/C Repeat
DREB: Dehydration-Responsive Element-Binding Proteins
DW: Dry Weight
EDTA: Ethylenediaminetetraacetic Acid
ENet: Efficient Neural Network
EIT: Electrical Impedance Tomography
EM: Electromagnetic
EVI: Extended Visual Information
FW: Fresh Weight
GBDT: Gradient Boosting Decision Trees
GBM: Gradient Boosting Machines
GPU: Graphical Processing Unit
H₂O₂: Hydrogen Peroxide
HPLC: High-Performance Liquid Chromatography
HSPs: Heat Shock Proteins
ICP-OES: Inductively Coupled Plasma Optical Emission Spectrometry
IPM: Integrated Pest Management
IRGAs: Infrared Gas Analyzers
IWM: Integrated Weed Management
JA: Jasmonic Acid
k-NN: K-Nearest Neighbor
LC-MS/MS: Liquid Chromatography Tandem Mass Spectrometry
LiDAR: Light Detection And Ranging
LIME: Local Interpretable Model-agnostic Explanations
LPO: Lipid Peroxidation
LSTM: Long Short-Term Memory
MDA: Malondialdehyde
ML: Machine Learning
NADPH: Nicotinamide Adenine Dinucleotide Phosphate
NBT: Nitroblue Tetrazolium
NDVI: Normalized Difference Vegetation Index
NIR: Near Infrared
NN: Neural Networks
NO: Nitrogen Oxides
NPQ: Non-Photochemical Quenching
NPQ: Non-Photochemical Quenching
PAM: Pulse Amplitude Modulated
PAR: Photosynthetically Active Radiation
PCA: Principal Component Analysis
Ph: Potential Of Hydrogen
POD: APX, Peroxidases
PRI: Photochemical Reflectance Index
PSII: Photosystem II
PVP: Polyvinylpyrrolidone
qPCR: Quantitative Real-Time Polymerase Chain Reaction
RD29A: Response-To-Dehydration 29a
RDI: Regulated Deficit Irrigation
REIP: Red Edge Inflection Point
ResNet: Residual Network
RF: Radiofrequency
RF: Random Forest
RFR: Random Forest Regression
RGB: Red Green And Blue
RL: Reinforcement Learning
RNA: Ribonucleic Acid
RNNs: Recurrent Neural Networks
ROS: Reactive Oxygen Species
RWC: Relative Water Content
SA: Salicylic Acid
SAR: Systemic Acquired Resistance
SDS-PAGE: Sodium Dodecyl Sulfate-Polyacrylamide Gel Electrophoresis
SHAP: Shapley Additive Explanations
SL: Supervised Learning
SLR: Simple Linear Regression
SO₂: Sulfur Dioxide
SOD: Superoxide Dismutase
SOM: Self-Organizing Map
SPAD: Soil Plant Analysis Development
SVMs: Support Vector Machines
SWIR: Shortwave Infrared
TBA: Thiobarbituric Acid
TBARS: Thiobarbituric Acid Reactive Substances
TCA: Trichloroacetic Acid
TRIzol: Total RNA Isolation Reagent
t-SNE: T-Distributed Stochastic Neighbor Embedding
TW: Turgid Weight
UAVs: Unmanned Aerial Vehicles
UN: United Nations
UV: Ultraviolet
UV-B: Ultraviolet B Radiation
ViT: Vision Transformer
WSNs: Wireless Sensor Networks
XGBoost: Extreme Gradient Boosting
IIoT: Industrial Internet Of Things
IoT: Internet Of Things
